# scEMAIL: Universal and Source-free Annotation Method for scRNA-seq Data with Novel Cell-type Perception

**DOI:** 10.1016/j.gpb.2022.12.008

**Published:** 2023-01-03

**Authors:** Hui Wan, Liang Chen, Minghua Deng

**Affiliations:** 1 School of Mathematical Sciences, Peking University, Beijing 100871, China; 2 Huawei Technologies Co., Ltd., Beijing 100080, China; 3 Center for Statistical Science, Peking University, Beijing 100871, China; 4 Center for Quantitative Biology, Peking University, Beijing 100871, China

**Keywords:** Cell-type annotation, Transfer learning, Privacy preservation, Single-cell RNA sequencing, Gene expression

## Abstract

Current **cell-type annotation** tools for **single-cell RNA sequencing** (scRNA-seq) data mainly utilize well-annotated source data to help identify cell types in target data. However, on account of **privacy preservation**, their requirements for raw source data may not always be satisfied. In this case, achieving feature alignment between source and target data explicitly is impossible. Additionally, these methods are barely able to discover the presence of novel cell types. A subjective threshold is often selected by users to detect novel cells. We propose a universal annotation framework for scRNA-seq data called scEMAIL, which automatically detects novel cell types without accessing source data during adaptation. For new cell-type identification, a novel cell-type perception module is designed with three steps. First, an expert ensemble system measures uncertainty of each cell from three complementary aspects. Second, based on this measurement, bimodality tests are applied to detect the presence of new cell types. Third, once assured of their presence, an adaptive threshold via manifold mixup partitions target cells into “known” and “unknown” groups. Model adaptation is then conducted to alleviate the batch effect. We gather multi-order neighborhood messages globally and impose local affinity regularizations on “known” cells. These constraints mitigate wrong classifications of the source model via reliable self-supervised information of neighbors. scEMAIL is accurate and robust under various scenarios in both simulation and real data. It is also flexible to be applied to challenging single-cell ATAC-seq data without loss of superiority. The source code of scEMAIL can be accessed at https://github.com/aster-ww/scEMAIL and https://ngdc.cncb.ac.cn/biocode/tools/BT007335/releases/v1.0.

## Introduction

With its rapid development, single-cell RNA sequencing (scRNA-seq) technology has become a powerful tool for comprehensive biological studies at an unparalleled high resolution [1]. Compared to traditional bulk gene expression analysis, which measures an average transcription level of bulk cells, scRNA-seq quantifies single-cell transcriptional profiling and identifies tissue heterogeneity at the cellular level [2]. With the sustained progress of sequencing technology and reduction of sequencing cost, scRNA-seq enables us to comprehensively sequence and annotate cell types existing in almost any tissue of any species [3], making it possible to identify unique biological functions of known or new cell types. Typical cell-type annotation methods involve three steps. The cell population is first clustered without supervision; and then specific marker genes for each cluster are found through differential expression analysis; finally cells are annotated according to their Gene Ontology functions [4]. However, for most clustering methods, it is hard to determine the cluster number in advance. Cell annotation based on marker genes also has drawbacks. First, the use of marker genes in different experiments varies widely, making uniformity challenging [5]. Manual literature review and database retrieval are quite challenging for non-specialists. Secondly, with the exponential growth of available datasets nowadays, this task becomes onerous, irreproducible, and time-consuming [6].

To fill this gap, automatic classification methods have emerged to annotate cells directly from scRNA-seq data, benefiting users without enough background knowledge. These methods utilize knowledge in well-annotated source datasets to assist cell-type identification in new target data. For example, scmap [7] assigns cell types by measuring the maximum similarity between known cluster centroids or cells in the source and target samples. Although efficient for large-scale data, it ignores the global structure of clusters and may suffer performance collapse owing to technical biases. Seurat v3 [8] explores the pairwise relationship between cells and constructs the shared nearest neighbor graph to identify cell types. However, when performing feature alignment, its computational burden is large, and specific cell-type information in the source data is neglected.

Recently, owing to the strength of deep learning in feature representation, deep neural networks have been utilized to realize cell label transfer. ItClust [9] is an iterative deep transfer learning-based model that trains well-annotated source data as the initialization to annotate target cells. Yet, training source data first and then fine-tuning the target clustering network may potentially risk ignoring the accumulated knowledge learned from source cells when training target data. In addition, ItClust does not consider local affinity constraints of similar cells, which often enhance cell clustering and annotation performance. To avoid the shortcomings of these methods, scSemiCluster [10] adds structural similarity regularization to the source data aiming to constrain clustering results of the target data. Pairwise constraints are further introduced on the labeled data to reinforce tightness and purity of clusters.

However, one practical issue that we should consider is the unavailability of source data. All the aformentioned methods require access to both source and target data throughout the training process for feature alignment and reduction of batch effect. Unfortunately, privacy preservation, safety issues, and storage limitations make this request impractical and inefficient in real applications. Data sharing can also be inhibited by legal restrictions if sensitive information is involved. As a new attempt, scArches [11] is a transfer learning strategy which maps target data on top of a source dataset by single-cell architectural surgery without sharing raw source data. It can be compatible with various autoencoder models such as scANVI [12] and trVAE [13]. However, the VAE-based models face a trade-off between the expression ability and the calculation cost of the variational posterior distribution. If the variational posterior distribution is relatively simple, it may not be able to approximate the true posterior distribution, resulting in a poor model effect; and if the selection is complicated, it will be difficult to optimize the network [14].

Besides, when target data contain novel cell types, it is also essential to accurately identify them. Existing methods usually cannot detect the existence of new cell types if no prior information is involved and if a clear rejection mechanism is not in place. Concretely, scmap (cluster-based) calculates the correlation scores between target cells and cluster centers in the source data. However, to annotate certain cells as “unknown”, it heavily relies on manually defined empirical thresholds, resulting in unstable results. ItClust only provides a confidence score for each cluster to annotate all cells in that cluster. Meanwhile, scSemiCluster measures prediction entropy to describe how likely a target cell is novel. Yet, neither ItClust nor scSemiCluster explicitly provides the selection scheme of subjective thresholds. Furthermore, research shows that the class competitive nature of deep learning-based methods may lead to overconfident predictions for true “unknown” cells, making the threshold hard to adjust [15], [16]. scArches first calculates the uncertainty of each target cell via nearest neighbors and then reports those with more than 50% uncertainty as “unknown”. Nevertheless, owing to the complexity of single-cell data and heterogeneity of various cell types, applying a uniform threshold for all annotation tasks is inflexible.

In view of two vital aspects discussed above, *i.e.*, source data accessibility and novel cell-type identification, we propose scEMAIL, a universal transfer learning-based annotation framework for scRNA-seq data, which incorporates expert ensemble novel cell-type perception and local affinity constraints of multi-order, with no need for source data. Generally, we first utilize source data to train the source classification model and then only apply this source model to annotate target data. For both source and target data training, a zero-inflated negative binomial (ZINB) model-based denoising autoencoder is introduced to depict the overall probabilistic structure of scRNA-seq data. To distinguish new cell types automatically without prior information, a novel cell-type perception module is designed. In detail, we first provide each target cell with an expert ensemble score (E-score) for uncertainty measurement combining three complementary indices. It is observed that their empirical distribution is bimodal or unimodal, largely reflecting the presence of new cell types or not. Hence, based on these measurements, we conduct the bimodality tests to judge whether a new cell type is present in the target data. If we confirm its existence, target cells are then separated into “known” and “unknown” groups based on an adaptive data-based threshold by manifold mixup. This data-partition task helps avoid error propagation or overconfident predictions of the network training that follows.

**Figure 1:**
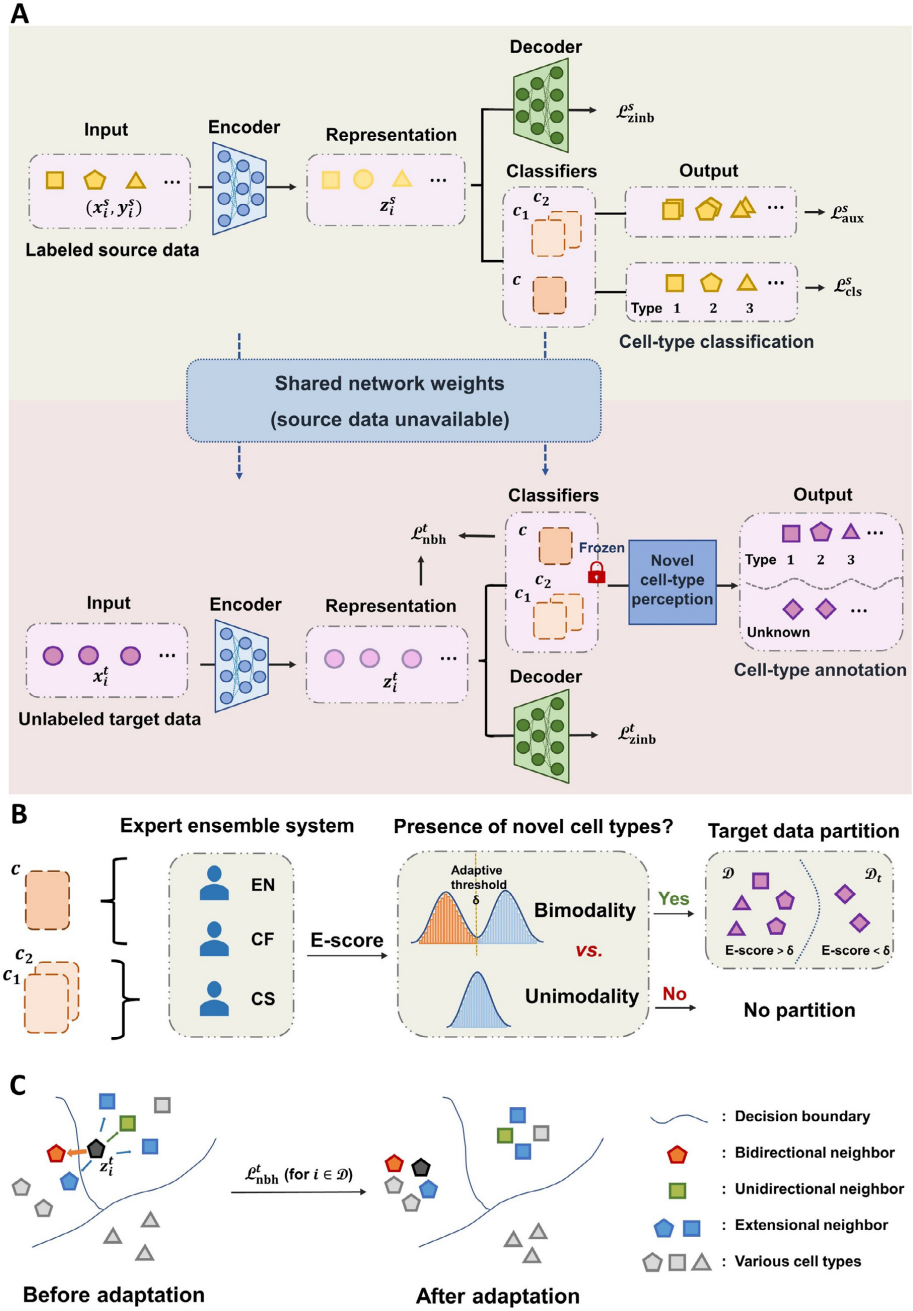
**Overview of the scEMAIL model** **A.** Supervised learning on well-labeled source data and model adaptation on unlabeled target data. The source model pre-training stage includes negative log-likelihood loss of ZINB distribution for data denoising and cross-entropy loss for supervised classification. We further introduce two different auxiliary classifiers in order for the following novel cell-type detection of target data. Once the pre-training process is finished, only the weights of pre-trained source model are offered to users freely without leaking the source data. Based on the outputs of source classifiers, a novel cell-type perception module is designed. It verifies the existence of novel cell types and partitions target data if novel cell types are discovered. At the model adaptation stage toward target data, the denoising ZINB loss and multi-order local affinity constraints are imposed to implicitly mitigate batch effect between source and target data. **B.** A novel cell-type perception module. We measure uncertainty of target cells via an expert ensemble system combining EN, CF, and CS. Based on the obtained E-scores, the bimodality of the empirical distribution is tested to verify the existence of novel cell types. If new cell types are discovered, an adaptive threshold partitions the target cells into “known” and “unknown” groups. **C.** The effect of multi-order local affinity constraints. To avoid the wrong classification by source model owing to the batch effect and encourage the prediction consistency of adjacent target cells, multi-order information of neighbors is gathered. We impose different levels of affinity constraints to a “known” target cell. Bidirectional neighbors are given larger weights than unidirectional and extensional neighbors. ZINB, zero-inflated negative binomial; EN, entropy; CF, confidence; CS, consistency.

To mitigate the impact of batch effect between source and target data with no raw source dataset at hand, we implement a self-supervised training scheme toward target cells during the model adaptation phase. According to the relative semantic relationship, we search for bidirectional, unidirectional, and extensional neighbors of each target cell in global scope. Based on the gathered multi-order neighborhood message, we impose various local affinity constraints on “known” target cells. This step fully utilizes the intrinsic relationship among target cells and alleviates wrong predictions of source model caused by batch effect. The overall framework of scEMAIL is displayed in Figure 1A. We conduct a large variety of simulation and real data experiments to verify the performance of scEMAIL. Under all possible scenarios, even when dealing with atlas-level datasets, cross-tissue datasets, as well as differentiation datasets, scEMAIL shows strong universality and competitive performance. It is worth mentioning that scEMAIL can be extended to other data types besides scRNA-seq data. Our additional experiments on the paired cellular indexing of transcriptomes and epitopes by sequencing (CITE-seq) data and single-cell sequencing assay for transposase-accessible chromatin (scATAC-seq) data, for example, confirm the flexibility and superiority of scEMAIL.

## Method

We begin with problem setting and notations. Suppose that the source data consist of $N_{s}$ well-annotated cells with gene expression vectors ${\left\{x_{i}^{s}\right\}}_{i=1}^{N_{s}}$ and labels ${\left\{y_{i}^{s}\right\}}_{i=1}^{N_{s}}$. The target data consist of $N_{t}$ cells ${\left\{x_{i}^{t}\right\}}_{i=1}^{N_{t}}$ with labels ${\left\{y_{i}^{t}\right\}}_{i=1}^{N_{t}}$ unobserved. Denote $T_{s}$ and $T_{t}$ as the label sets of the source and target datasets, respectively. Then $T=T_{s}\cap T_{t}$ is the common label set shared by both source and target data, while $\overset{¯}{T_{s}}=T_{s}\backslash T$ and $\overset{¯}{T_{t}}=T_{t}\backslash T$ are the label sets exclusive, or “private”, to source and target, respectively. According to the affiliation of these two label sets, we will encounter the following four cases. If target label set exactly equals the source label set, *i.e.*, $\overset{¯}{T_{s}}=\overset{¯}{T_{t}}=\emptyset$, we term this case as “closed setting”; if the target cell types are a subset of source cell types, *i.e.*, $\overset{¯}{T_{t}}=\emptyset$, but $\overset{¯}{T_{s}}\neq \emptyset$, this is termed as “partial setting”; contrarily, if $\overset{¯}{T_{s}}=\emptyset$, but $\overset{¯}{T_{t}}\neq \emptyset$, we term this as “open setting”, because the target label set contains both source label set and novel (also termed as “target private”) cell types; finally, the most challenging but realistic, setting is one where the target data contain a subset of the source cell types, as well as some novel cell types, *i.e.*, $\overset{¯}{T_{s}}\neq \emptyset$ and $\overset{¯}{T_{t}}\neq \emptyset$, which we term as “open-partial setting”.

The lack of target labels makes it impossible to prejudge which of the four cases will contain our data. Previous methods usually place emphasis on closed or partial setting, resulting in rather poor performance under other settings. Here, our goal is to achieve satisfactory and stable performance under all settings. That is, besides accurately annotating target cells in T, if any target samples belong to $\overset{¯}{T_{t}}$, we should also distinguish these “unknown” cells. Besides, considering the privacy issue and storage constraint, we assume that we can only access the source data during source model training since availability will vanish thereafter. Instead, a pre-trained source model could be offered to users freely for application to various target datasets without leaking the source data.

### Network construction of source model

The basic source model can be divided into data denoising and classification. Here, inspired by deep count autoencoder [17], we first utilize ZINB distribution to model scRNA-seq data. Denote the expression of the j-th gene in the i-th source cells as $x_{\mathrm{ij}}^{s}\;\left(1\leq i\leq N_{s},1\leq j\leq p\right)$. ZINB distribution consists of zero-inflation parameter $\omega_{\mathrm{ij}}^{s}$ (the probability of dropout events), mean parameter $\mu_{\mathrm{ij}}^{s}$, and dispersion parameter $\phi_{\mathrm{ij}}^{s}$:

(1) $$\begin{array}{c} \begin{array}{c} P_{\text{zinb}}\left(x_{\mathrm{ij}}^{s}|\omega_{\mathrm{ij}}^{s},\mu_{\mathrm{ij}}^{s},\phi_{\mathrm{ij}}^{s}\right)=\omega_{\mathrm{ij}}^{s}\delta_{0}\left(x_{\mathrm{ij}}^{s}\right)+\left(1-\omega_{\mathrm{ij}}^{s}\right)\frac{\Gamma \left(x_{\mathrm{ij}}^{s}+\phi_{\mathrm{ij}}^{s}\right)}{\Gamma \left(x_{\mathrm{ij}}^{s}+1\right)\Gamma \left(\phi_{\mathrm{ij}}^{s}\right)}\\ {\left(\frac{\phi_{\mathrm{ij}}^{s}}{\phi_{\mathrm{ij}}^{s}+\mu_{\mathrm{ij}}^{s}}\right)}^{\phi_{\mathrm{ij}}^{s}}{\left(\frac{\mu_{\mathrm{ij}}^{s}}{\phi_{\mathrm{ij}}^{s}+\mu_{\mathrm{ij}}^{s}}\right)}^{x_{\mathrm{ij}}^{s}} \end{array} \end{array}$$

Then, we adopt a ZINB model-based autoencoder to reveal the global probabilistic structure of scRNA-seq data. In detail, after being projected into latent representation by encoder h, the estimations of three sets of parameters are outputted through a decoder g. To reconstruct data, we use negative log-likelihood of ZINB distribution as loss function:

(2) $$\begin{array}{c} \begin{array}{c} L_{\text{zinb}}^{s}=-\frac{1}{N_{s}\cdot p}\sum_{i=1}^{N_{s}}\sum_{j=1}^{p}\log P_{\text{zinb}}\left(x_{\mathrm{ij}}^{s}|\omega_{\mathrm{ij}}^{s},\mu_{\mathrm{ij}}^{s},\phi_{\mathrm{ij}}^{s}\right) \end{array} \end{array}$$

We suppose the length of $T_{s}$ is K, $y_{i}^{s}\in \left\{1,2,\cdots ,K\right\}$. After projecting cell $x_{i}^{s}$ into $z_{i}^{s}$, a classifier c takes $z_{i}^{s}$ as input and outputs a soft probability vector $p_{i}^{s}$ of K-dimension where the k-th element $p_{i,k}^{s}$ is the assignment probability that the i-th cell belongs to the k-th cell type. We first train source data with ground-truth labels by minimizing the cross-entropy loss:

(3) $$\begin{array}{c} \begin{array}{c} L_{\text{cls}}^{s}=-\frac{1}{N_{s}}\sum_{i=1}^{N_{s}}\sum_{k=1}^{K}q_{i,k}\log p_{i,k}^{s} \end{array} \end{array}$$

where $q_{i,k}$ is the one-of-K encoding of $y_{i}^{s}$*.*

Different from an ordinary source model, we further introduce two auxiliary classifiers $c_{j}$, *j* = 1, 2, which also take features from h for further uncertainty measurement (see the next section). The k-th element of their output auxiliary soft probability vectors is denoted as $p_{i,k}^{s,j}$, *j* = 1, 2. Hence, we train these two auxiliary heads of source model by minimizing the co-training supervised classification function:

(4) $$\begin{array}{c} \begin{array}{c} L_{\text{aux}}^{s}=-\frac{1}{N_{s}}\sum_{j=1}^{2}\sum_{i=1}^{N_{s}}\sum_{k=1}^{K}q_{i,k}log\;p_{i,k}^{s,j} \end{array} \end{array}$$

To maintain the diversity of different classifiers, we do not update the parameter weights of $c_{j}$ and initialize $c_{j}$ with different random initial values. We can obtain the total target function for source model training:

(5) $$\begin{array}{c} \begin{array}{c} L^{s}=L_{\text{zinb}}^{s}+L_{\text{cls}}^{s}+L_{\text{aux}}^{s} \end{array} \end{array}$$

In practice, we can replace raw one-hot labels with smooth labels to increase model discriminability (File S1).

### Novel cell-type perception

Existing methods ignore the necessity of training “unknown” and “known” groups separately and do not identify the existence of novel cell types, which is sub-optimal for network training. On the one hand, if we force the partitioning of cells into a “novel” group when no unknown cell types can be found in the target data, it will lead to error propagation and accumulation during network training, resulting in poor annotation results. On the other hand, if we assume no novel cell types during the training process, when the fact is just the opposite, the network will produce overconfident predictions in the absence of novel cell-type detection. Therefore, after the source model completes pre-training, the aforementioned considerations call for the ability to separate target cells belonging to $\overset{¯}{T_{t}}$ from those belonging to T before we utilize the trained source model to fit the target data. To address this requirement, we herein introduce a novel cell-type perception module. The outline view of this module is shown in Figure 1B.

#### Expert ensemble system for uncertainty measurement

To detect novel cell types in the target data, it can be reasonably assumed that data in target private label set $\overset{¯}{T_{t}}$ has more uncertainty than target data in common label set T. Entropy is the most commonly used uncertainty measurement. However, as elaborately explained in the study by Fu and colleagues [18], when the prediction is extremely confident, or close to uniform, entropy is insensitive to probability changes. Therefore, it may fail to distinguish extremely sharp and highly uncertain predictions. As a complement, confidence performs high discriminability for highly uncertain and confident predictions. Hence, it is necessary to combine entropy and confidence together in order to produce an accurate measurement for both smooth and sharp cell-type distributions. However, their correctness of uncertainty measurement has an underlying premise. That is, the batch effect between the source and target data is negligible. If not, the source classifier may potentially risk producing an overconfident prediction error of a novel cell. In this case, both entropy and consistency will falsely recognize it as a “known” cell. To compensate for this shortcoming, we introduce consistency based on two different auxiliary classifiers during source model training to indicate their agreement of predictions. The lower the consistency of predictions, the more possible a target cell is novel. Consistency is robust to prediction errors caused by the batch effect, as it is less likely that both classifiers will wrongly predict a cell into exactly the same cell type.

In conclusion, to define a comprehensive and suitable uncertainty measurement, we adopt an expert ensemble system including three components: entropy (EN), confidence (CF), and consistency (CS). Each has its own trait and cannot represent uncertainty by itself. In detail, the entropy and confidence of a target cell by classifier c are defined as:

(6) $$\begin{array}{c} \begin{array}{c} \text{E}\text{N}\left(z_{i}^{t}\right)=-\sum_{k=1}^{K}p_{i,k}^{t}\log p_{i,k}^{t},\;\;\;\;\;\;\;\;\text{C}\text{F}\left(z_{i}^{t}\right)=\underset{k=1,\cdots ,K}{\max}p_{i,k}^{t} \end{array} \end{array}$$

where $z_{i}^{t}$ is the corresponding latent representation of $x_{i}^{t}$, and $p_{i,k}^{t}$ is the k-th element of the K-dimensional prediction vector $p_{i}^{t}$ outputted by c. The consistency of prediction is defined as:

(7) $$\begin{array}{c} \begin{array}{c} \text{C}\text{S}\left(z_{i}^{t}\right)=p_{i}^{t,1}\cdot p_{i}^{t,2} \end{array} \end{array}$$

where $p_{i}^{t,j}$, *j* = 1, 2 are the auxiliary soft probability vectors of two auxiliary classifiers $c_{j}$, *j* = 1, 2. We further divide logK in order to $\text{EN}(z_{i}^{t})$ to ensure that it is within the range [0,1]. The final E-score for uncertainty measurement is defined as:

(8) $$\begin{array}{c} \begin{array}{c} \text{E}\text{-}\text{s}\text{c}\text{o}\text{r}\text{e}\left(z_{i}^{t}\right)=\frac{1-\text{EN}\left(z_{i}^{t}\right)/log\;K+\text{CF}\left(z_{i}^{t}\right)+\text{CS}\left(z_{i}^{t}\right)}{3} \end{array} \end{array}$$

A higher score increases the certainty that a cell belongs to a known cell type.

#### Test for the existence of novel cell types

**Figure 2:**
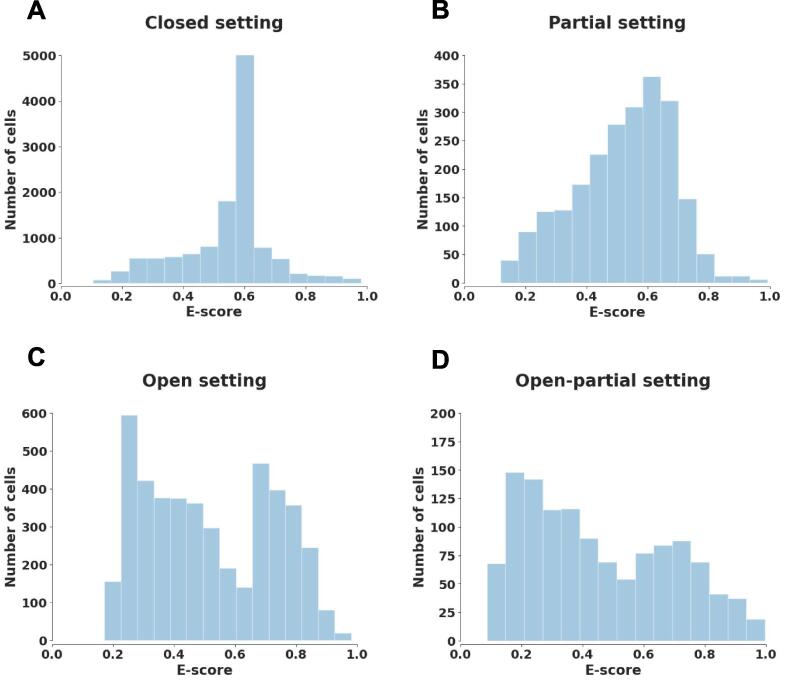
**Empirical distributions of E-scoresbefore adaptation for four datasets** **A.** Dataset “bone marrow” [34] under closed setting. **B.** Dataset “pancreas” [35] under partial setting. **C.** Dataset “mammary gland” [3] under open setting. **D.** Dataset “neonatal rib” [34] under open-partial setting.

When target data have novel cell types, it is interesting that their empirical distribution of ${\left\{\text{E}\text{-}\text{score}(z_{i}^{t})\right\}}_{i=1}^{N_{t}}$ exhibits apparent bimodal structure when compared to target data with no unknown cells (Figure 2). This phenomenon can be explained by the overall distribution difference of E-scores between known and unknown groups. Here we adopt two different measures of bimodality, the bimodality coefficient [19] and Hartigan’s dip test [20] (see File S1 for detailed definitions), to determine the bimodality of the obtained empirical distribution. As long as one of these measures indicates the bimodal structure of data distribution, we will suppose the existence of novel cell types in the following training process.

#### Adaptive data-based threshold by manifold mixup

If the bimodality tests suggest no target private cell types, we can suppose our task is under closed or partial setting and skip this threshold selection step. Otherwise, it is always a challenging task to decide a proper threshold of uncertainty to accurately separate target cells from T and those from $\overset{¯}{T_{t}}$. Motivated by a previous study [21], to simulate novel cell types with no deterministic patterns, we herein construct synthetic manifolds by arbitrarily mixing up two target cells, as follows:

(9) $${\overset{∼}{z}}_{i,j}^{t}=\lambda_{i,j}z_{i}^{t}+\left(1-\lambda_{i,j}\right)z_{j}^{t},\;i\neq j,\;\lambda_{i,j}∼\text{Uniform}\left(0,1\right)$$

The final threshold can be adaptively and automatically decided by taking the average E-score of all possible pairs of the target cells, as follows:

(10) $$\delta =\frac{1}{N_{t}\left(N_{t}-1\right)}\text{E}\text{-}\text{score}\left({\overset{∼}{z}}_{i,j}^{t}\right),\;\forall i\neq j$$

This threshold selection scheme is based on the assumption that by mixing two different representations randomly, irrespective of whether their labels are known or not, we will get a relatively novel representation [22]. The adaptive choice of threshold δ is in line with our intuition because if a small group of cells belongs to $\overset{¯}{T_{t}}$, then δ will be smaller than the majority of $\text{E}\text{-}\text{score}\left(z_{i}^{t}\right),\;i=1,\cdots ,N_{t}$, while the relative value of δ will increase if the proportion of cells in $\overset{¯}{T_{t}}$ is larger. Thus, with a convincing threshold, we partition the target data via the following rule:

(11) $$\begin{array}{c} \left\{\begin{array}{c} i\in D,\;\text{i}\text{f}\;\;\text{E}\text{-}\text{s}\text{c}\text{o}\text{r}\text{e}\left({\text{z}}_{i}^{t}\right)>\delta \\ i\in D_{t},\;\;\;\text{o}\text{t}\text{h}\text{e}\text{r}\text{w}\text{i}\text{s}\text{e} \end{array}\right),\;\forall i=1,\cdots ,N_{t} \end{array}$$

where D and $D_{t}$ are the indices of target cell subsets that we suppose to possess “known” and “novel” labels, respectively. We separate target data into “known” and “unknown” groups so that the following network adaptation can be more directed and targeted. More simply, if tests for bimodality indicate no novel cell types, subset D is defined as the total $N_{t}$ indices of target data ${\left\{x_{i}^{t}\right\}}_{i=1}^{N_{t}}$, while $D_{t}$ is an empty set.

### Model adaptation to the target data

After uncertainty measurement by the expert ensemble system and target data partition, we should fully utilize cells from D to mitigate batch effect between source and target data. Since source data are not accessible during adaptation, the only message that we could utilize is the intrinsic structure relationship within target data. Owing to the distribution shift caused by batch effect, the source model may classify adjacent target cells into various cell types. We believe that cells of similar representations should be in the same cluster with a high probability. Hence, we utilize multi-order message of neighbors to avoid the wrong supervision of source model. Based on the neighborhood information that we gather, different levels of local affinity constraints are imposed to a target sample to encourage the prediction consistency of adjacent cells, as shown in Figure 1C.

Denote the dimension of features in the latent space as d. To search for neighbors in global scope during batch training, we first maintain two memory banks: representation bank $R_{N_{t}\times d}={\left(z_{i}^{t},\cdots ,z_{N_{t}}^{t}\right)}^{T}$ and soft label bank $S_{N_{t}\times K}={\left(p_{i}^{t},\cdots ,p_{N_{t}}^{t}\right)}^{T}$. These two offline banks are proposed to preserve current representations and predictions of target data without the involvement of gradient propagation. For a representation $z_{i}^{t}$, we can first retrieve the indices of its M-nearest neighbors from bank R based on cosine similarity:

(12) $$\begin{array}{c} \begin{array}{c} V_{M}^{i}=\underset{j}{{\mathrm{argtop}}_{M}}\left(\pi \left(z_{i}^{t},R_{j}\right)|j=1,\cdots ,N_{t}\right) \end{array} \end{array}$$

where the operation $\underset{j}{{\mathrm{argtop}}_{M}}\left(f\left(R_{j}\right)|j=1,\cdots ,N_{t}\right)$ extracts the indices from $1,\cdots ,N_{t}$ that gives the M largest value of a target function $f\left(R_{j}\right)$, $\pi \left(x,y\right)=\frac{x\cdot y}{\left\|x\|\|y\right\|}$, and $R_{j}$ is the j-th element of bank R.

Note that we deliberately include the target sample itself in its nearest neighbors, which can be regarded as a self-regularization step to avoid drifting far away from current predictions and decrease the potential effect of noisy neighborhoods. Then, according to the semantic relationship, we can divide the neighbors of each cell into three categories: bidirectional, unidirectional, and extensional. In detail, among the nearest neighbors of $z_{i}^{t}$, some also regard $z_{i}^{t}$ as their nearest neighbors, while some do not. We surmise that the relationship between $z_{i}^{t}$ and the former is much closer. Therefore, we define the index set of bidirectional neighbors (also known as mutual nearest neighbors) $V_{1}^{i}$ and unidirectional neighbors $V_{2}^{i}$ as follows:

(13) $$\begin{array}{c} V_{1}^{i}=\left\{j|j\in V_{M}^{i}\wedge i\in V_{M}^{j}\right\},\;V_{2}^{i}=V_{M}^{i}\backslash V_{1}^{i} \end{array}$$

Though it is straightforward to set a large value of M to collect more neighbor information, this strategy risks the introduction of “bad” neighbors belonging to different cell types. Instead, we consider the neighbors of each nearest neighbor to aggregate more local information. The index set of these extensional neighbors can be defined as:

(14) $$\begin{array}{c} \begin{array}{c} V_{3}^{i}=\left\{l|l\in V_{M}^{j},j\in V_{M}^{i}\right\} \end{array} \end{array}$$

These extensional neighbors incorporate high-order intrinsic information of target data. Compared with considering more nearest neighbors, they are anticipated to be more intimate with the target data on the manifold [23]. Among these three types of neighbors, we believe that bidirectional neighbors have a higher potential to belong to the same cell type as the i-th cell does; therefore, we assign them with larger affinity values than those of the other neighbors. To prevent the negative effect of noisy neighbors in $D_{t}$, we only preserve neighbors in D. The overall optimizing function for local affinity constraints is:

(15) $$\begin{array}{c} \begin{array}{c} L_{\text{nbh}}^{t}=-\frac{1}{\left\|D\right\|}\sum_{i\in D}\left(\sum_{j\in V_{1}^{i}\cap D}S_{j}^{T}+\alpha \left(\sum_{k\in V_{2}^{i}\cap D}S_{k}^{T}+\sum_{l\in V_{3}^{i}\cap D}S_{l}^{T}\right)\right)p_{i}^{t} \end{array} \end{array}$$

where ‖D‖ is the cell number in D, $p_{i}^{t}$ is the soft label of the i-th target data in D, and $S_{j}$ is the j-th element of S. The default value of α is 0.1.

We also add the denoising ZINB loss for target data with the same notation meaning as Equation (2), as:

(16) $$\begin{array}{c} \begin{array}{c} L_{\text{zinb}}^{t}=-\frac{1}{N_{t}\cdot p}\sum_{i=1}^{N_{t}}\sum_{j=1}^{p}\log P_{\text{zinb}}\left(x_{\mathrm{ij}}^{t}|\omega_{\mathrm{ij}}^{t},\mu_{\mathrm{ij}}^{t},\phi_{\mathrm{ij}}^{t}\right) \end{array} \end{array}$$

To preserve the distribution message of unseen source data, we fix the parameter weights of source classifiers and only fine-tune the encoder h. The final objective of knowledge adaptation from pre-trained source model to target data is

(17) $$\begin{array}{c} \begin{array}{c} L^{t}=L_{\text{zinb}}^{t}+L_{\text{nbh}}^{t} \end{array} \end{array}$$

As illustrated before, for the latter target function $L_{\text{nbh}}^{t}$, if we suppose the existence of unknown cell types, we only train subset D of the target samples with high certainty measurements to reduce overconfident predictions on novel cell types. D and $D_{t}$ are updated every iteration during model adaptation. Once training is finished, for cells in D, the final annotation label will be determined by the maximum component of classifier c; for cells in $D_{t}$, they will be annotated as “unknown”. The entire workflow of scEMAIL is displayed in Algorithm 1 and more implementation details can be seen in File S1.

**Figure:**
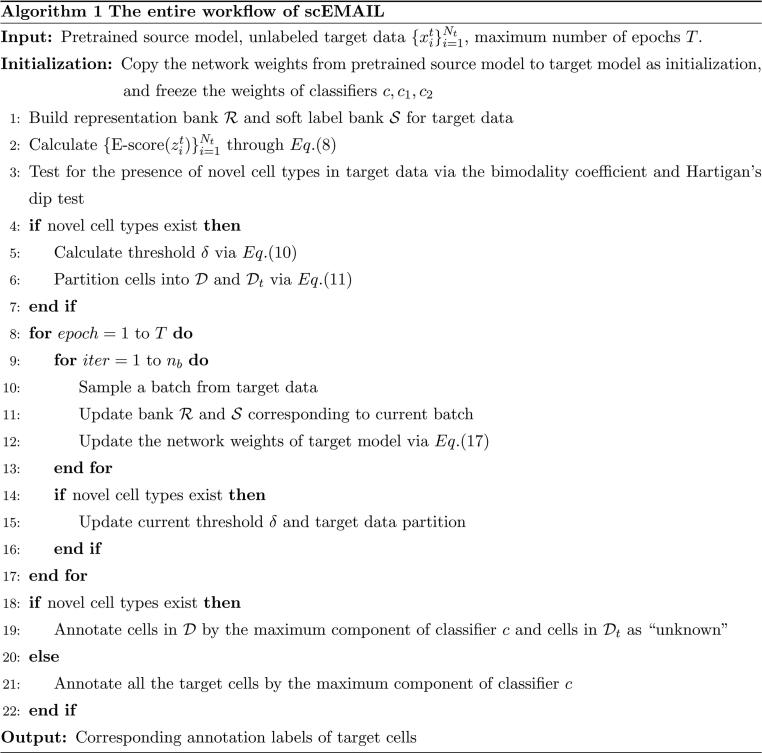

## Results and discussion

To comprehensively evaluate the performance of scEMAIL under various biological scenarios, both simulation and real data experiments were performed with all possible settings considered. We compared scEMAIL with five state-of-the-art models: Seurat v3, scmap (cluster-based), ItClust, scSemiCluster, and scArches (based on scANVI). Among them, scArches does not need access to target data during adaptation, similar to scEMAIL. ItClust trains source and target data separately, which is somewhat similar to the source-free setting. We also recorded the results if applying the source model to the target data directly without fine-tuning (denoted as “source only”). For datasets of close or partial settings, *i.e.*, where novel cell types are absent, we calculated annotation accuracy over all target cells. For those of open or open-partial settings (*i.e.*, novel cell types exist), all target private cells are assumed to form a single “unknown” cell type. Besides total annotation accuracy, the trade-off between the accuracy of “known” and “novel” cells is also essential. Hence, the H-score, which calculates the harmonic mean of the accuracy on “known” and “unknown” cells, is also adopted to evaluate the performance of all methods [18]. The implementation details of competing methods can be seen in Table S1.

Our main experimental results include eight subsections. The first and second subsections are the basic experiments. By dividing the simulated and real datasets into the predefined four settings, we comprehensively compared the performance of the models in all the possible scenarios. The third subsection is the ablation study, which respectively tested the performance gain brought by the main innovation components of scEMAIL. The fourth, fifth, and sixth subsections introduce more diverse and challenging annotation tasks, which applied atlas-level data with mixed batches, cross-tissue data with limited overlap of cell types, and differentiation data with continuous variation information. In the last two subsections, we further extended our model framework to other omics data. scEMAIL was applied to the paired CITE-seq data and scATAC-seq data, demonstrating its potential on multi-omics data. Other more experimental results are presented and analysed in detail in File S1.

### scEMAIL maintains satisfactory performance under four settings in simulated experiments

In terms of dataset size, we considered two basic simulated situations. One is “larger”, which means that we set the sample size of source data to be twice that of the target. The other situation is “smaller”, in which the size of source data was set to be half that of the target. In terms of the relationship of source and target label spaces, the simulation experiments were divided into four parts: closed setting, partial setting, open setting, and open-partial setting. For closed setting, we examined the effect of different cluster numbers on annotation accuracy. For the latter three settings, we defined three indices to measure the degree of imbalance between source and target label spaces: “partialness” for partial setting, “openness” for open setting, and “privateness” for open-partial setting. Details about simulated experiments are shown in File S1. The higher each index is, the less shared label space we set between source and target datasets.

**Figure 3:**
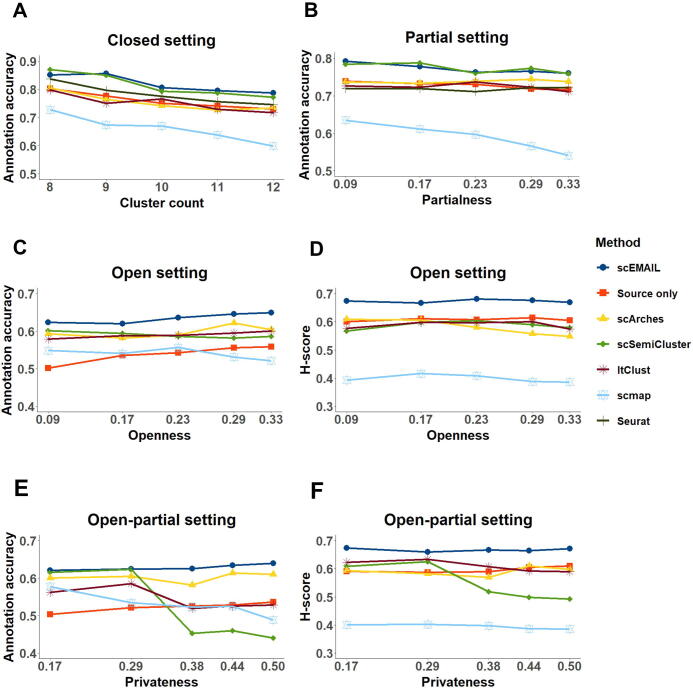
**Line graphs of simulation experiments in “larger” situation** Line graphs of total annotation accuracy under four settings with the variation of cluster count (**A**), “partialness” (**B**), “openness” (**C**), and “privateness” (**E**). The H-scores are also provided for open (**D**) and open-partial (**F**) settings.

In Figure 3, we showed the performance of each model under four settings in the “larger” situation. For the closed setting, as the cluster numbers increased, the accuracy of all models diminished. scEMAIL, scSemiCluster, and Seurat were superior to baseline “source only” regardless of cluster numbers, while ItClust, scArches, and scmap were underperforming. For the partial setting, scmap was mostly affected by the degree of “partialness”. scEMAIL and scSemiCluster also had a slight downward trend, but they generally maintained satisfactory performance among the compared methods. For open and open-partial settings with “unknown” cells included, the preponderance of scEMAIL was more obvious, which confirmed its stability and accuracy on both known cell-type annotation and novel cell detection. The performance of scArches was relatively steady with mild fluctuations. And the total accuracies of scSemiCluster, ItClust, and scmap were higher than that of baseline when the “unknown” proportion was small, essentially because the cost of falsely recognizing “unknown” cells was slight at this time. As the degree of “openness” and “privateness” increased, their advantages over baseline gradually decreased. In the sense of H-score, which can reflect the trade-off of accuracies between “known” and “unknown” cells, as shown in Figure 3, the performance of scmap and scSemiCluster was flawed. Scmap was too weak to identify new cell types resulting in a low H-score, and the accuracy of scSemiCluster droped sharply under the open-partial setting, which may result from the mixing of “known” and “unknown” cell types during training and the overlapping of their uncertainty measurement. We can reach similar conclusions for experiments in the “smaller” situation from Figure S1.

### scEMAIL balances known cell-type annotation and novel cell-type detection under four settings in real data experiments

For real data analysis, we adopted 8 pairs of scRNA-seq datasets generated from different organisms and organs (Table S2). Each pair of datasets was named after their corresponding organs. For each experiment, we ran each method with 10 random seeds and recorded the median value. In Table 1, we showed the total annotation accuracies for the first four experiments under closed or partial settings, while for the latter four experiments under open or open-partial settings, the reported total accuracies and H-scores were exhibited. We can observe that scEMAIL performed best among all competing methods with all annotation accuracies greater than 0.7. scSemiCluster and scArches showed relatively satisfactory results. They performed better than “source only” baseline in the first four experiments, while the performance of ItClust was unstable, highly relying on the selected datasets. However, these three deep learning-based algorithms tended to produce overconfident predictions on novel cell types, which made it difficult for them to differentiate between “known” and “unknown” cell types when compared to scEMAIL. In addition, ItClust potentially risked the misalignment of cell labels and clusters, resulting in rather low annotation accuracy (dataset “placenta”) or H-score (dataset “peripheral blood”). This is mainly because the knowledge and the label information of the source data may be ignored during the model adaptation process. scEMAIL, however, could effectively avoid this problem by fixing the source classifiers during model adaptation. Seurat also performed well under closed and partial settings. However, it lacked the ability to recognize novel cell types, which severely limited its use in real data. Besides, the result of scmap was unfulfilling. It was very sensitive toward threshold selection, but susceptible to technical noise. Overall, scEMAIL was robust under various settings. It could effectively transfer knowledge learned from source model to target data, and it possessed high discriminability when novel cell types existed.

**Table 1:** **Annotation accuracy of each method ineight pairs of scRNA-seq datasets**

**Setting**	**Dataset**	**Seurat**	**scmap**	**ItClust**	**scSemiCluster**	**scArches**	**Source only**	**scEMAIL**
Closed	Placenta	0.7422	0.6567	0.1219	0.7982	0.6151	0.5903	**0.8065**
	Bone marrow	0.8551	0.5093	0.8564	0.8498	0.8436	0.8388	**0.8873**
Partial	Pancreas	0.9465	0.9461	0.9420	0.9548	0.9443	0.9356	**0.9676**
	Trachea	0.9348	0.5978	0.9230	0.9288	0.9104	0.8793	**0.9415**
Open	Mammary gland	/	0.9431 (0.9259)	0.8041 (0.7653)	0.8259 (0.8328)	0.8070 (0.8148)	0.9168 (0.9169)	**0.9830** **(0.9794)**
	Lung	/	0.5743 (0.5543)	0.4083 (0.5195)	0.7349 (0.5846)	0.7107 (0.7345)	0.6853 (0.7792)	**0.7822** **(0.8619)**
Open-partial	Neonatal rib	/	0.3648 (0.2397)	0.5916 (0.5902)	0.6803 (0.6737)	0.7105 (0.6644)	0.7962 (0.7940)	**0.9162** **(0.9140)**
	Peripheral blood	/	0.2119 (0.1653)	0.5925 (0.0215)	0.5069 (0.4105)	0.6238 (0.5338)	0.6573 (0.6698)	**0.7387** **(0.7622)**

*Note*: Each pair of datasets is named after their corresponding organs. For the last four pairs of datasets with novel cell types, we also provide the H-scores in parentheses. Highest score for each pair of datasets is in bold. scRNA-seq, single-cell RNA sequencing.

**Figure 4:**
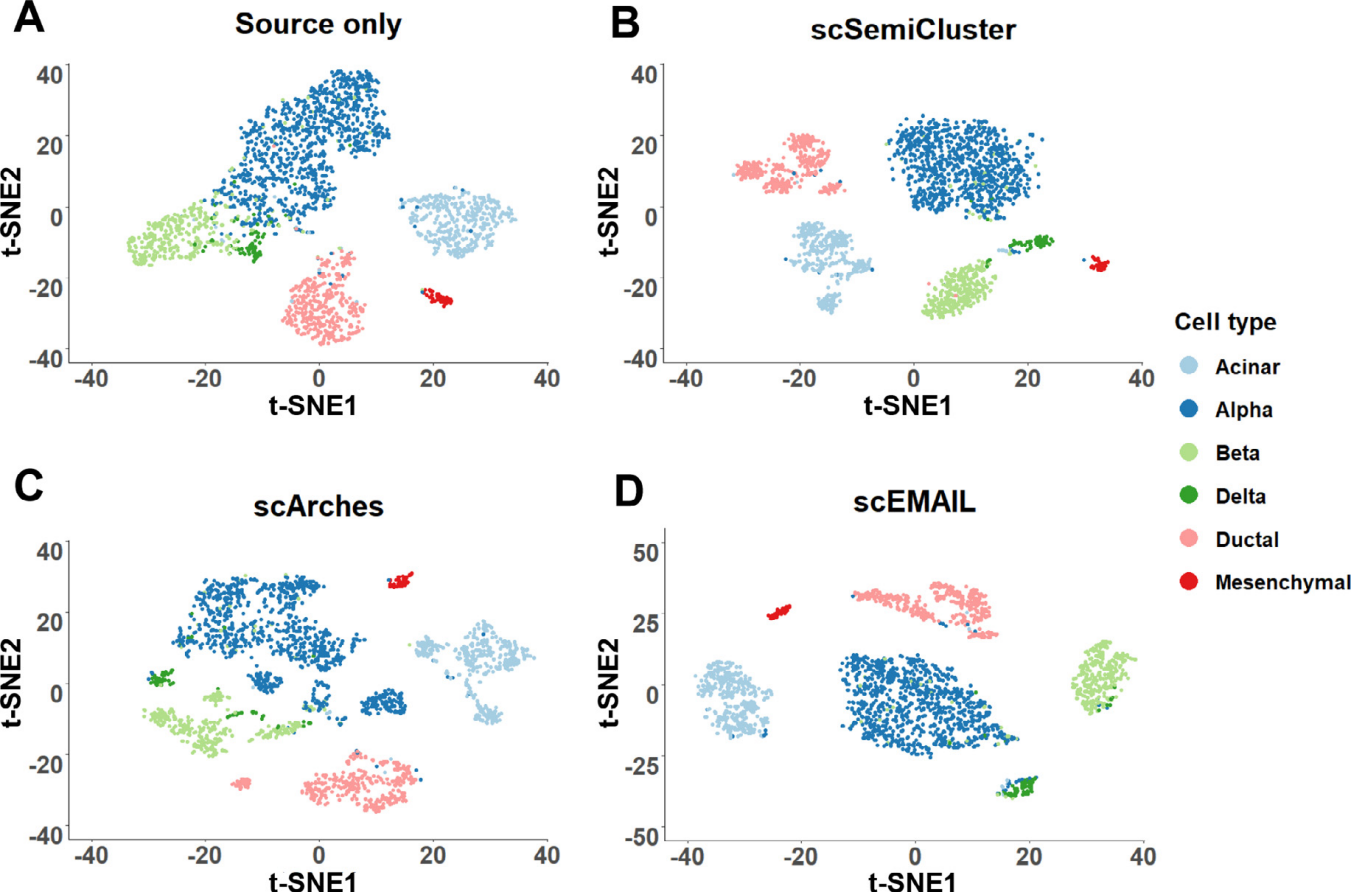
**Visualization plots of cell-type detectionbyfour compared methodsfordataset “pancreas” via t-SNE** Dataset “pancreas” [35] is under open setting. We exhibit the 2-dimensional t-SNE projection of the latent representations from source model only (**A**), scSemiCluster (**B**), scArches (**C**), and scEMAIL (**D**). t-SNE, t-distributed stochastic neighbor embedding.

We also showed the two-dimensional visualization plots to compare the clustering results of scEMAIL, scSemiCluster, and scArches intuitively. For each method, we extracted and visualized the low-dimensional representations by t-distributed stochastic neighbor embedding (t-SNE). For the task of datasets “pancreas” under partial setting, as shown in Figure 4, we could observe that both scEMAIL and scSemiCluster can clearly discriminate among different cell types, but scSemiCluster tended to separate the cell types “acinar” and “ductal” into several clusters. Furthermore, the latent space learned from scArches failed to form correct and distinct cluster structure. Cells of cell types “alpha”, “beta”, and “ductal” were scattered in the t-SNE plot of scArches, which was not desirable. Compared with baseline model “source only” which mixes up cell types “alpha”, “beta”, and “delta” in the latent space, scEMAIL leveraged the intrinsic relationship of target data through local consistency constraints and, hence, can distinguish these three cell types after model adaptation.

To clearly demonstrate the alignment relationship of different cell types, we further used Sankey plots to display the annotation results of the pair of “mammary gland” datasets in Figure S2. The target dataset of this task preserved three novel cell types, including “B cell”, “T cell”, and “macrophage”, which should be annotated as “unknown”. For cells of both “known” and “unknown” types, scEMAIL can find their correct correspondence, confirming our claims that multi-order affinity constraints benefit the classification of “known” cells, while the novel cell-type perception module significantly helps in new cell-type detection. However, the three other methods all mistakenly assigned “unknown” labels to a subset of “endothelial cell” and “stromal cell”. Furthermore, scSemiCluster and scArches falsely annotated a certain proportion of target private types as “luminal epithelial cell” or “basal cell”. This phenomenon results from the fact that their models confuse “known” and “unknown” cell types, resulting in overlapping measurements between the two and poor discrimination during novel class recognition. The aforementioned results reconfirm the superiority of scEMAIL.

### Ablation study on the major contributing components of scEMAIL

In this section, we conducted several ablation studies to verify the validity of three main innovative contributions introduced in scEMAIL, respectively. First is the E-score measurement of uncertainty to identify novel cell types. Second is the multi-order local affinity constraints to mitigate batch effect. Last is target data partition during model adaptation to avoid error accumulation and overconfident predictions.

#### Ablation study on expert ensemble measurement of uncertainty

We further verified whether gathering three complementary measurements of uncertainty is superior to only using one or two evaluation scores in Figure S3. Among all the candidates, the overall performance of expert ensemble measurement was best. Sometimes, using one or two evaluation scores could also reach satisfactory results, but each score had its own limitations and cannot behave well in all scenarios. For example, for datasets “mammary gland” and “peripheral blood”, consistency can significantly improve performance. It may be because of larger batch effect existing in these datasets, which makes the prediction errors of entropy and confidence larger. Hence, a more robust and comprehensive uncertainty measurement scheme is the E-score obtained by combining these three uncertainties.

#### Ablation study on neighbor affinity constraints

We first explored the effect and accuracy gain of three kinds of neighbors, respectively. As shown in Figure S4, we observed that considering all neighbors can lead to better performance, but that the removal of any one of them may decrease model accuracy by different degrees. This proves the rationality of introducing prediction consistency of neighbors. Specially, we observed that bidirectional neighbors were important and brought annotation improvement, which is in line with our assumption that they are likely to belong to the same cluster as the target cell. In addition, unidirectional and extensional neighbors also provided additional supervised information and benefited the network to enhance discriminability.

#### Ablation study on target data partition

We then studied the effectiveness of training local affinity constraints $L_{\text{nbh}}^{t}$ in subset D, instead of the entire set. As shown in Figure S5, if novel cell types exist, partitioning target data can increase classification accuracy. This is possible because forcing an “unknown” sample to preserve consistent prediction with its neighbors may introduce noise and result in overconfident output.

**Figure 5:**
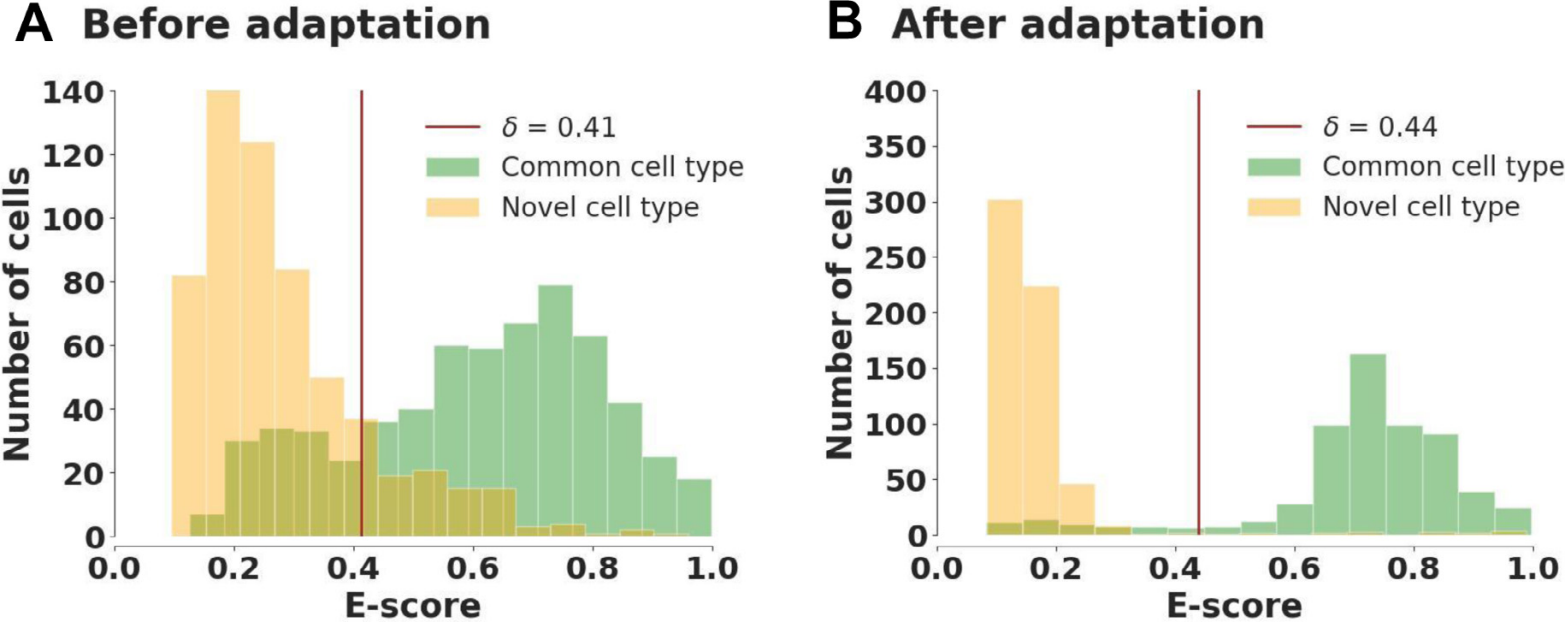
**Distribution difference based on E-scores before and after model adaptation of dataset “neonatal rib” via frequency histograms** Dataset “neonatal rib” [34] is under open-partial setting with one target private cell type. The plots are drawn before (**A**) and after (**B**) model adaptation. The red vertical lines are the adaptive thresholds given by manifold mixup.

To visually present the rationality of threshold selection and data partition, in Figure 5 we showed the division between “known” and “unknown” cells before and after adaptation. Before model adaptation, a number of target cells were misclassified owing to an overlap between “known” and “unknown” sample distributions, even though a suitable threshold was given. Through data partition during training, the E-scores of these two kinds of cells were completely separated, making it easy to find those cells that preserve novel labels. We also conducted robustness experiments on two hyper-parameters introduced in our model: number of nearest neighbors M and relative affinity value α. The performance of scEMAIL was stable no matter the variation of both hyper-parameters. Besides, we have performed scalability experiments to explore the running time and memory usage of each annotation method on large-scale datasets. Robustness analysis of adaptive threshold via manifold mixup was also conducted. These experimental results can be found in File S1.

### scEMAIL can deal with atlas-level datasets with mixed batches

**Figure 6:**
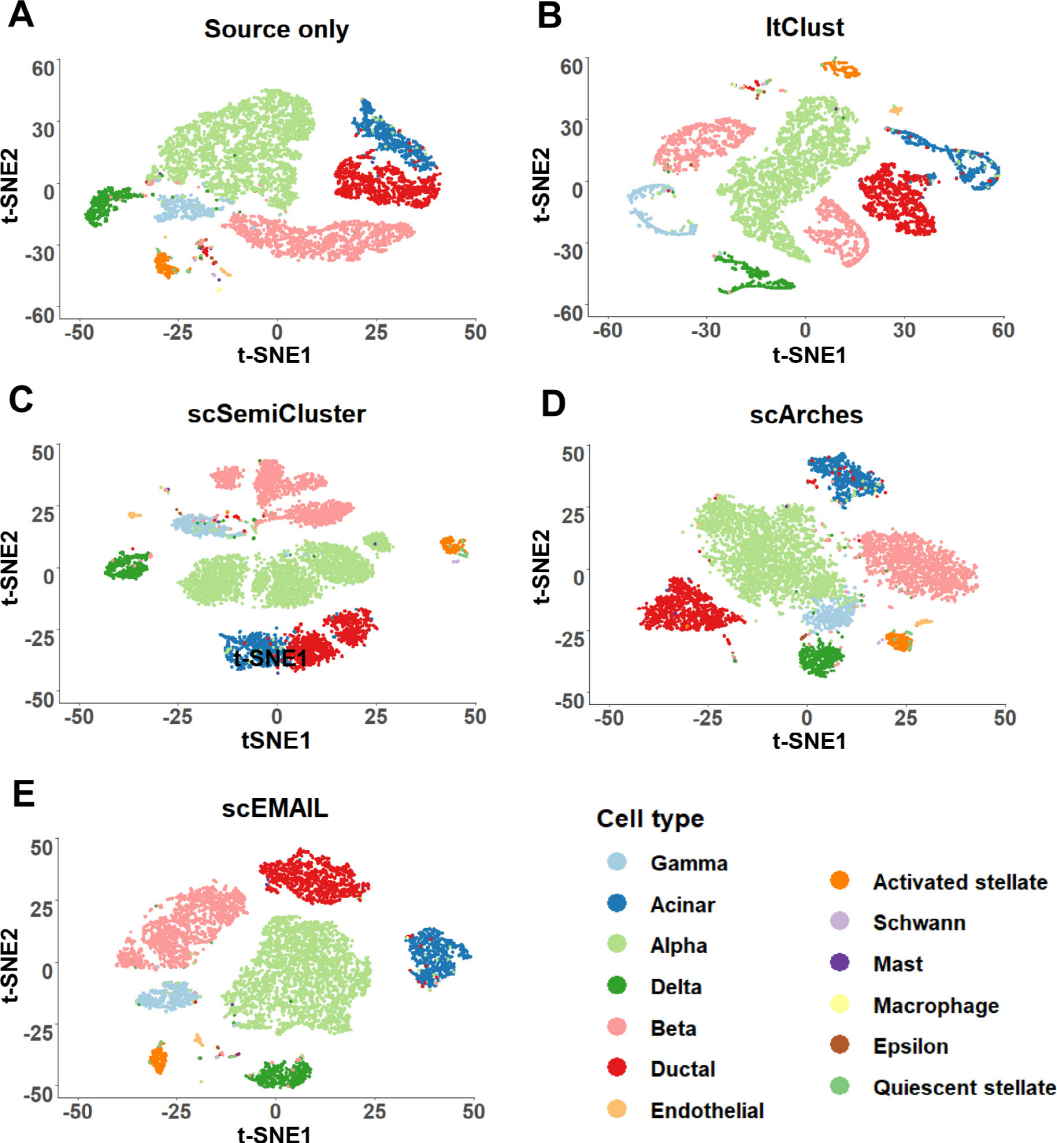
**Visualization plots of cell-type detectionbyfive compared methods for the atlas-level “human pancreas (full)” task via t-SNE** The atlas-level “human pancreas” dataset is obtained from [24]. We exhibit the 2-dimensional t-SNE projection of the latent representations from source model only (**A**), ItClust (**B**), scSemiCluster (**C**), scArches (**D**), and scEMAIL (**E**).

We additionally conducted a series of experiments on two atlas-level scRNA-seq datasets which can be regarded as benchmarking [24]. These tasks were challenging because both source and target data were mixed with multiple batches, which placed higher demands on the ability of algorithms to handle complex batch effects. To compare our models more widely, we further recorded the results of three additional popular algorithms SingleR [25], CHETAH [26], and SingleCellNet [27] on all these atlas-level annotation tasks. Since SingleCellNet does not provide functions to detect unknown cell types, we only recorded its results on tasks without unknown cell types in the target data. The first dataset “human immune” consisted of 10 different batches. For the “human immune (subset)” task, source and target data contained 2 and 4 different batches, respectively. Based on this, 2 additional batches were incorporated into the source and target data separately to obtain the “human immune (full)” task (Tables S3 and S4). For both tasks, four novel cell types existed in the target data, and the annotation accuracy and H-score of each method are exhibited in Table 2. We obeserved that the performance of scArches and scEMAIL were relatively satisfactory, while the performance of scmap on the full task exhibited a certain degree of decline, having difficulty in handling mixed batches well. Although ItClust had a high overall annotation accuracy on the full task, it and scSemiCluster had relatively low H-scores, reflecting their lack of ability to identify new cell types. The total annotation accuracy of SingleR was satisfactory, but its ability to identify new cell types was less competitive. The second dataset was the “human pancreas”, including 9 different batches. For the “human pancreas (subset)” task, the source and target data contained 3 and 4 batches, respectively. For the “human pancreas (full)” task, the source data contained 4 batches and the target data contained the remaining 5 batches (Tables S3 and S4). For these two tasks, there was no novel cell type in the target data, thus we only showed the total annotation accuracy of each algorithm in Table 2. scEMAIL exhibited superior performance on both tasks. In addition, the annotation accuracy of scmap, ItClust, and scSemiCluster were relatively higher, while the performance of Seurat and scArches was worse. To further visualize whether the latent representations obtained by deep learning-based methods have clear cluster structures, we presented their t-SNE visualization plots for the “human pancreas (full)” task in Figure 6. It can be seen that scSemiCluster separated cells of the same cell type into several sub-clusters, which made the clusters too scattered. ItClust also mistakenly divided cell type “delta” and “beta” into two parts, which was not a desirable cluster structure. In comparison, the latent space learned from scArches could achieve relatively compact structures, but different cell types were not dispersed enough, for example, there was no obvious distinction between cell type “alpha” and “gamma”. The cluster structure obtained by scEMAIL was satisfactory. Compared with the “source only” model without an adaptation process, scEMAIL achieved intra-cluster compactness and inter-cluster separation, which indicated that the affinity constraints guide the network to learn the correct intercellular relationships.

**Table 2:** **Annotation accuracy of each method for 6 atlas-level scRNA-seq annotation tasks**

**Task**	**Seurat**	**Single-CellNet**	**SingleR**	**CHETAH**	**scmap**	**ItClust**	**scSemiCluster**	**scArches**	**Source only**	**scEMAIL**
Human immune (subset)	/	/	**0.7154** (0.6383)	0.5555 (0.5611)	0.6408 (0.6189)	0.6352 (0.6185)	0.4138 (0.4178)	0.5901 (0.6314)	0.6308 (0.6780)	0.7015 **(0.7164)**
Human immune (full)	/	/	0.7347 (0.6331)	0.6387 (0.6484)	0.5525 (0.5691)	0.7028 (0.5153)	0.5046 (0.4737)	0.6409 (0.6803)	0.6572 (0.7338)	**0.7473** **(0.7811)**
Human pancreas (subset)	0.8714	0.9249	0.9568	0.9082	0.9533	0.9589	0.9358	0.8883	0.9165	**0.9680**
Human pancreas (full)	0.8972	0.9053	0.9612	0.9241	0.9601	0.9617	0.9018	0.8952	0.9077	**0.9674**
Human pancreas (source data excluded “alpha”)	/	/	0.6071 (0.6066)	0.5669 (0.5367)	0.7413 (0.7196)	0.6745 (0.6711)	0.3718 (0.3408)	0.6519 (0.6574)	0.8595 (0.8673)	**0.9641** **(0.9650)**
Human pancreas (source data excluded “acinar”)	/	/	0.5387 (0.3423)	0.5307 (0.4163)	**0.7808** (0.6493)	0.7362 (0.1929)	0.6556 (0.3159)	0.5981 (0.5035)	0.7089 (0.6931)	0.7536 **(0.8356)**

*Note*: For the tasks with novel cell types, we also provide the H-scores in parentheses. Highest score for each task is in bold.

**Figure 7:**
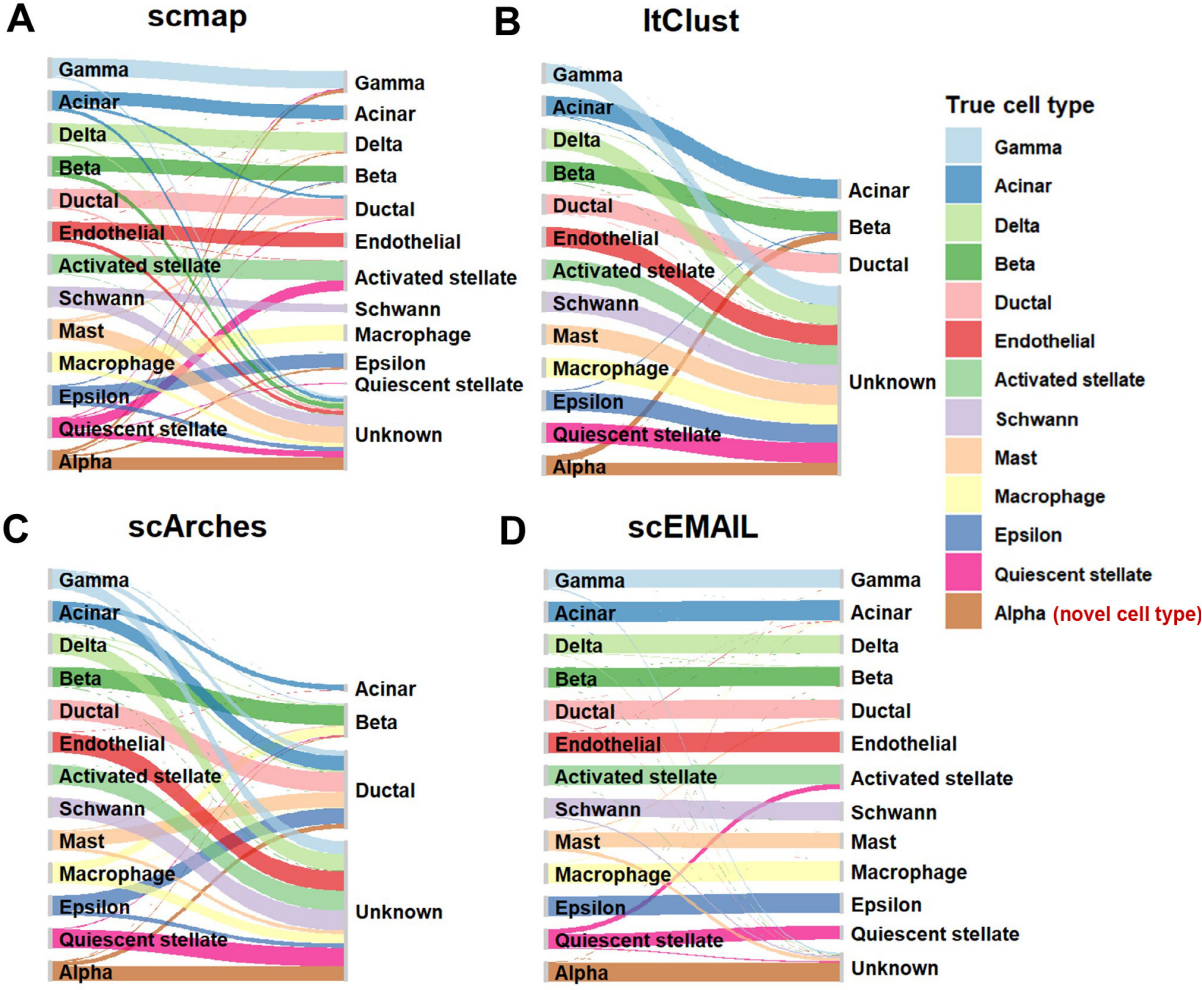
**Mapping between real cell types and predicted cell types** by **four compared methodsfordataset “human pancreas” via Sankey plots** The atlas-level “human pancreas” dataset is obtained from [24]. For this task, cell type “alpha” is artificially removed in the source data. The annotation results of scmap (**A**), ItClust (**B**), scArches (**C**), and scEMAIL (**D**) are exhibited.

To further explore the ability of each method to identify certain novel cell types, we artificially removed cell types “alpha” and “acinar” in the source data based on the “human pancreas (full)” task, making them private to the target data, respectively, thus constituting two additional cell annotation tasks. As shown in Table 2, the performance of scSemiCluster, ItClust, and scArches in identifying new cell types was not stable enough. These three deep learning-based methods confused “known” and “unknown” cell types during training, making it hard to distinguish novel cell types. The performance of CHETAH and SingleR was less competitive in terms of both total annotation accuracy and H-score. scEMAIL performed well in recognizing new cell types with H-scores above 0.8 for both tasks. We further exhibited the Sankey diagram of scEMAIL, scArches, ItClust, and scmap on the task with the novel cell type “alpha” in Figure 7. It can be seen that scmap, ItClust, and scArches all recognized a certain proportion of known cells, such as “mast” and “macrophage”, as “unknown”, resulting in a much higher proportion of identified unknown cell types than the real situation; in addition, ItClust and scArches also falsely annotated a proportion of cell type “alpha” as a “known” type. On the contrary, scEMAIL could find almost completely accurate correspondence, correctly classifying cell type “alpha” as “unknown”, and not erroneously classifying other “known” cell types into this category. Overall, although more popular models were considered and compared, scEMAIL still exhibited advantages and showed desirable performance.

### scEMAIL can be applied on cross-tissue experiments

To test the performance of the source model when fitted to target data from different tissues, we applied the *Tabula Muris* Consortium dataset [3] profiled mouse cells using Smart-seq2 (SS2) and 10× Genomics to conduct two sets of cross-tissue experiments. The first group of experiments took the data derived from mammary gland using 10× Genomics sequencing technology as the source data to train a source model, and then provided this source model to the target data derived from fat and limb muscle using SS2 technology, respectively. The second group of experiments utilized source data derived from limb muscle using 10× Genomics sequencing technology and adapted it to the target data derived from kidney and diaphragm using SS2, respectively. The corresponding experimental results are shown in Table 3. Detailed biological and statistical information about these four annotation tasks can be viewed in Table S5.

**Table 3:** **Annotation accuracy of each method on cross-tissue experiments**

**Source tissue**	**Target tissue**	**scmap**	**ItClust**	**scSemiCluster**	**scArches**	**Source only**	**scEMAIL**
Mammary gland	Fat	0.2158 (0.1997)	0.5702 (0.5661)	0.4752 (0.4388)	0.7340 (0.6262)	0.7152 (0.7273)	**0.7358** (**0.7619**)
	Limb muscle	0.5890 (0.5952)	**0.7725** (0.7003)	0.5092 (0.3050)	0.6431 (0.4797)	0.5495 (0.5733)	0.7009 (**0.7352**)
Limb muscle	Kidney	0.7341 (0.2840)	0.7110 (0.7307)	0.8343 (0.7799)	**0.8786** (0.8063)	0.8430 (0.8405)	0.8651 (**0.8667**)
	Diaphragm	0.6080 (0.5838)	0.8241 (0.8051)	0.4299 (0.4083)	0.8713 (0.8731)	0.8144 (0.8171)	**0.8839** (**0.8918**)

*Note*: For tasks corresponding to the same source tissue, scEMAIL applies the same pre-trained source model on different target data. We exhibit the total annotation accuracy, and the H-scores are also provided in parentheses. Highest score for each task is in bold.

We observed that for the challenging cross-tissue annotation experiments, no method achieved the best performance on all tasks. Overall, the performance of scEMAIL was relatively robust, even in the case of low overlap between source data and target data (for example, when source tissue is limb muscle and target tissue is kidney, there are only 2 overlap cell types, but source private and target private cell types are 4 and 3, respectively). Compared with the source model only, our model adaptation process significantly improved the accuracy of model. The performance of scArches was also ideal, showing competitive performance on the first, third, and fourth tasks. ItClust had outstanding performance on the second task, and had achieved satisfactory results on other tasks. The performance of scmap and scSemiCluster was relatively unstable, and they encountered difficulty in aligning the same source data with the target data of different other organizations.

We also visualized the annotated results of the cross-tissue experiments with source tissue limb muscle and target tissue diaphragm by Sankey diagrams in Figure S6. In this task, the source data included six cell types, of which only three were shared with the target, and the target data also contained two novel cell types “lymphocyte” and “skeletal muscle satellite cell”. It can be seen that under this challenging circumstance, all methods had difficulty in identifying the novel cell type “lymphocyte”, which may be due to its small sample size, only 81, and high similarity with “B cell” in low-dimensional representations. In addition, except for scEMAIL, other methods also misclassified a large number of “known” cell types as “unknown”, resulting in lower annotation accuracy.

In conclusion, our method can be applied across tissues and brings an improvement of annotation performance within the possible range. However, for the selection of source data, although it is not limited to a specific tissue, we do not recommend using tissues that are too different from the target data as source data. If the cell types of them overlap too little, most of the target cells are novel, and only a small portion of cells can be annotated. It is contrary to our original intention that cell annotation is regarded as the basic goal, and novel cell-type detection is an additional task. Our source model can be used as a general pre-training information to assist annotating various target datasets. In addition, matching source data is beneficial to generate a good pre-training model and provide a reliable initialization during target model adaptation.

### scEMAIL can reach satisfactory annotation and novel cell-type detection results on differentiation data

The continuous differentiation process of cells in organisms generates new cell types, and thus differentiation data are a very realistic and challenging application for identifying novel cell types. In order to verify the performance of our method on the developmental or differentiation data, we have applied two differentiation datasets. The first differentiation dataset was from the adult human testis [28]. The differentiation process of sperm cells obeys the following precedence relationship of pseudotime: spermatogonial stem cells (SSC), differentiating S'gonia, early primary S'cytes, late primary S'cytes, round S'tids, elongated S'tids, and sperm. We retained these seven sperm differentiation lineage-associated cell types to test our model. The source data were derived from donors 1 and 2, and the target data were derived from donor 3. We artificially removed the most differentiated cell type “sperm” in the source data as the novel cell type of target. The results of each model on this task are shown in Table 4. Despite the continuum and similarities between different cell types, our method was able to identify differences in similar cells, while it also showed strong recognition ability in recognizing the most differentiated novel cell type “sperm”. The performance of scmap was relatively satisfactory, while other methods have not shown ideal performance for this challenging task. We also exhibited Sankey diagrams to intuitively show the annotation performance of each method in Figure S7. It can be seen that, except for scEMAIL, other methods tended to assign a portion of novel cell type “sperm” to the most similar cell type “elongated S'tids”. They also falsely annotated some cell types close to cell type “sperm”, such as “late primary S'cytes”, “round S'tids”, and “elongated S'tids”, as “unknown” cells. Compared to them, our method was less prone to such misplaced annotation results.

**Table 4:** **Annotation accuracy of each method on experiments ofdifferentiationdata**

**Tissue**	**Novel cell type**	**scmap**	**ItClust**	**scSemiCluster**	**scArches**	**Source only**	**scEMAIL**
Testis	Sperm	0.8383 (0.8389)	0.3278 (0.2174)	0.5782 (0.5142)	0.5459 (0.4983)	0.8931 (0.8925)	**0.9495** **(0.9505)**
Mammary gland	Avd	0.6719 (0.4267)	0.0043 (0.0036)	0.4897 (0.4287)	0.7876 (0.8083)	0.8364 (0.8071)	**0.9325** **(0.9372)**
	Hsd	0.5951 (0.4734)	0.3757 (0.0489)	0.3238 (0.3197)	0.7501 (0.7245)	0.8689 (0.8730)	**0.8760** **(0.8814)**

*Note*: For each task, we artificially remove the most differentiated cell type, *i.e.*, cell type “sperm”, “Avd”, and “Hsd”, respectively, to test the ability of each model when applying on differentiation data. We exhibit the total annotation accuracy, the H-scores are also provided in parentheses. Highest score for each task is in bold. Avd, differentiated alveolar cells; Hsd, hormone sensing differentiated.

**Figure 8:**
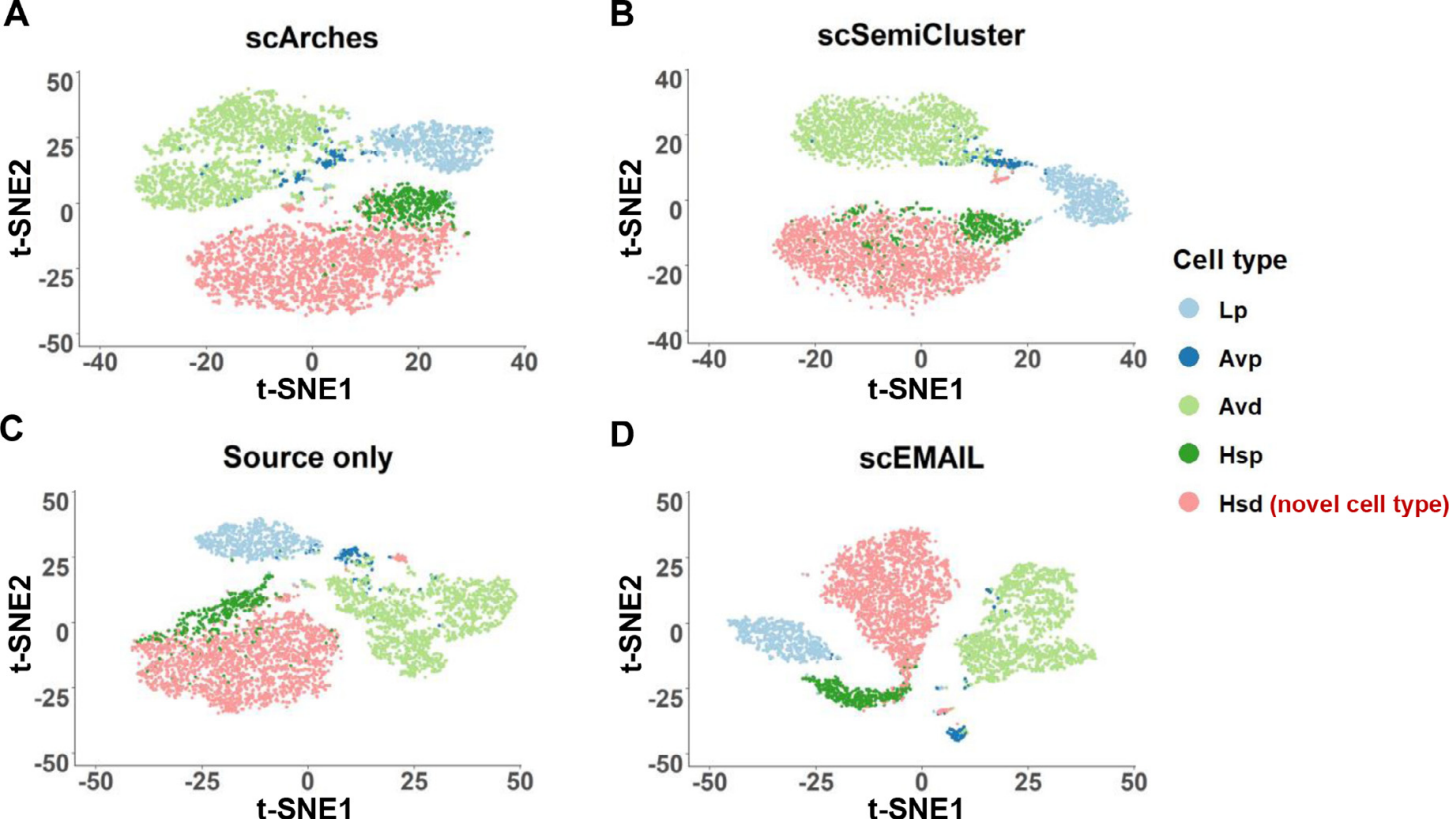
**Visualization plots of cell-type detectionbyfour compared methodsforthedifferentiationdataset “mammary gland” via t-SNE** The differentiation dataset “mammary gland” is derived from [29]. For this task, the most differentiated cell type “Hsd” is artificially removed in the source data. We exhibit the 2-dimensional t-SNE projection of the latent representations from scArches (**A**), scSemiCluster (**B**), source model only (**C**), and scEMAIL (**D**). Lp, luminal progenitor; Avp, progenitor alveolar cells; Avd, differentiated alveolar cells; Hsp, hormone sensing progenitors; Hsd, hormone sensing differentiated.

The second dataset was the differentiation data of mouse mammary gland epithelial cells [29]. Unlike the first dataset, its differentiation lineage has two branches, namely secretory alveolar lineage: luminal progenitor (Lp), progenitor alveolar cells (Avp), and differentiated alveolar cells (Avd); and hormone sensing lineage: Lp, hormone sensing progenitors (Hsp), and hormone sensing differentiated (Hsd). We retained these five cell types contained within these two lineage branches to conduct our experiments. The source data were derived from donors 1–5, and the target data were derived from donors 6–8. We artificially removed the most differentiated cell type “Avd” and “Hsd” in the source data as the novel cell type of target, respectively. The experimental results are shown in Table 4. The performance of scmap on these two tasks was slightly decreased, while the annotation accuracy of scArches was relatively better. scEMAIL maintained its superiority and could accurately identify these two novel cell types, respectively. We visualized the clustering effect of different methods on identifying the novel cell type “Hsd” by t-SNE in Figure 8. It can be seen that except for scEMAIL, other methods cannot accurately distinguish novel cell type “Hsd” from the highly similar cell type “Hsp”. Besides, scArches and scSemiCluster confused the high alike cell types “Avp” and “Avd” on another lineage branch, and cannot form clear boundaries between clusters. However, scEMAIL can better separate different cell types and annotate them accurately.

Last but not the least, we need to clarify that our network design has the effect of discretizing the continuous difference of differentiation data. In detail, we minimize the cross-entropy loss for cells with different cell-type labels in the pre-training stage, and the affinity constraints in the model adaptation stage promotes a distinct cluster structure. These manipulations can maximize the separation of individual cell types and identify differences between them, which is beneficial for clustering and classification tasks. However, if our ultimate goal is not just classification, and we also hope to retain the continuity information of the data to a certain extent, then some more customized network design may be needed (such as discussion by cases and judging in advance whether the data are of continuous difference [30]). In this way, we can reach a balance between retaining continuous information and maintaining the classification effect. This is a very interesting question, which is worth studying in our future work.

### The performance of scEMAIL can be enhanced by the complementary information of other omics data

Simply using scRNA-seq data for novel cell-type identification may be insufficient due to the noise of data or the lack of information. Leaving aside the constraints of data availability, the introduction of other omics or cell morphology can complement each other to reflect cellular heterogeneity from different perspectives.

In order to verify whether other omics data can assist scRNA-seq data in cell-type annotation and novel cell-type identification, we applied paired data from 10x 10 k peripheral blood mononuclear cell (PBMC) dataset, which was downloaded from 10x Genomics website (https://www.10xgenomics.com/resources/datasets/10-k-pbm-cs-from-a-healthy-donor-gene-expression-and-cell-surface-protein-3-standard-3-0-0), which provided additional phenotypic information by integrating measurements of cellular proteins and transcriptomes of single cells. A total of 7865 cells from a healthy donor were stained with 14 TotalSeq^TM^-B antibodies. Cell-matched scRNA-Seq data were also available. For the five major cell types in the dataset, including B cells, CD14^+^ monocytes, CD4^+^ T cells, CD8^+^ T cells, and natural killer (NK) cells, we conducted leave-one-out experiments, *i.e.*, artificially removing one cell type in the source data at one time and then keeping it as a novel cell type in the target data. The remaining four cell types were equally divided into the source and target data. The corresponding results of applying scRNA-seq data solely (single-omics) and applying both scRNA-seq data and cellular surface proteins (multi-omics) are shown in Table 5. For multi-omics experiments, the network input of a sample was obtained by simply preprocessing and standardizing the measurements of two omics separately and then concatenating them together. From the table we can see that for all the five experiments, the performance of scEMAIL has been significantly improved after the measurements of surface proteins were incorporated into the network input, and the H-score has increased by two to six percentage points. This is strong evidence that other omics data can supply information of cellular heterogeneity which is not detected in scRNA-seq data. Moreover, in the case that the network input remains unchanged, we can also conclude that the model adaptation step of scEMAIL can significantly improve the annotation accuracy compared to the results of source model only. For example, for the experiments with novel cell type “CD14^+^ monocytes”, scEMAIL can outperform the pre-trained source model by 15 and 10 percentage points in the single-omics and multi-omics experiments, respectively.

**Table 5:** **Comparisonof model performance for single-omics and multi-omics experiments on paired data from 10xGenomics10 k PBMC dataset**

**Novel cell type**	**Single-omics** **(scRNA-seq)**		**Multi-omics** **(scRNA-seq + cellular surface proteins)**	
	**Source only**	**scEMAIL**	**Source only**	**scEMAIL**
B cells	0.8552 (0.8837)	0.8652 (0.8933)	0.9126 (0.9192)	0.9163 (0.9225)
NK cells	0.8523 (0.8704)	0.9088 (0.9316)	0.9016 (0.9195)	0.9441 (0.9590)
CD14^+^ monocytes	0.7830 (0.7932)	0.9369 (0.9454)	0.8604 (0.8700)	0.9621 (0.9677)
CD4^+^ T cells	0.8647 (0.8528)	0.8881 (0.8895)	0.8978 (0.8916)	0.9321 (0.9339)
CD8^+^ T cells	0.8190 (0.7937)	0.8641 (0.8496)	0.8614 (0.8530)	0.9203 (0.9164)

*Note*: The 10x Genomics 10 k PBMC dataset consists of five different cell types. We conducted leave-one-out experiments, keeping one novel cell type at one time. The remaining four cell types are partitioned equally to form our source and target data. The corresponding results of applying scRNA-seq data solely and applying both scRNA-seq data and cellular surface proteins, respectively, are recorded. We exhibit the total annotation accuracy, and the H-scores are also provided in parentheses. NK, natural killer; PBMC, peripheral blood mononuclear cell.

**Figure 9:**
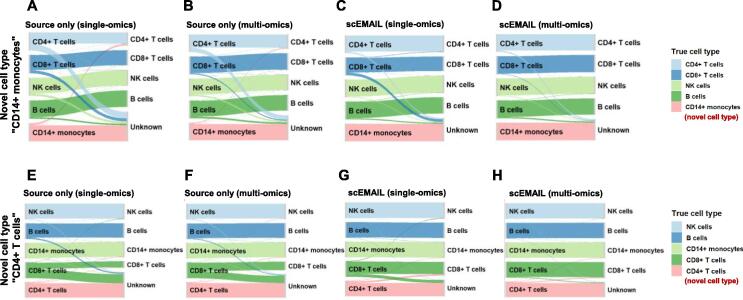
**Mapping between real cell types and predicted cell types on single-omics and multi-omics experiments via Sankey plots** These two tasks are applied on the paired data from 10x Genomics 10 k PBMC dataset downloaded from 10x Genomics website (https://www.10xgenomics.com/resources/datasets/10-k-pbm-cs-from-a-healthy-donor-gene-expression-and-cell-surface-protein-3-standard-3–0-0) with cell types “CD14^+^ monocytes” and “CD4^+^ T cells” set as novel cell types, respectively. For experiments with novel cell type “CD14^+^ monocytes”, we show the annotation results of source model only on single-omics (**A**) and multi-omics (**B**), as well as the results of scEMAIL on single-omics (**C**) and multi-omics (**D**) data. For experiments with novel cell type “CD4^+^ T cells”, the corresponding results of source model only on single-omics (**E**) and multi-omics (**F**), as well as the results of scEMAIL on single-omics (**G**) and multi-omics (**H**) data, are also exhibited. NK, natural killer; PBMC, peripheral blood mononuclear cell.

We displayed some visualization plots to show the improvement of the model due to the multi-omics data. In Figure 9, we used Sankey diagrams to show the annotation results of experiments with “CD14^+^ monocytes” and “CD4^+^ T cells” as novel cell types, respectively. Adding surface protein data can help us better classify cell types “CD4^+^ T cells” and “CD8+ T cells”, which may be due to the fact that its supplementary information can provide more detailed and differentiated information about the cell phenotype characteristics of these two cell types. We also presented t-SNE visualization plots for the task with novel cell type “CD8^+^ T cells” in Figure S8. Similarly, we found that the information introduced by multi-omics data obviously helped to distinguish between cell types “CD4^+^ T cells” and “CD8^+^ T cells” that were highly similar and thus originally mixed together. Although scEMAIL roughly classified the two cell types through self-supervised learning (Figure S8C), the clustering structure was obviously clearer (Figure S8D).

The popularity of single-cell multi-omics analysis in biological research has developed our comprehension of cellular heterogeneity. For example, proteins are known to be much more abundant than RNA and functionally directly participate in the process of cell–cell interactions and cell signalling [31]. The incorporation of cell surface proteins data has the potential to reveal cellular heterogeneity missed by single-modality scRNA-seq data and greatly expand the upper limit of our performance. These comparisons between single-omics and multi-omics experiments have given us good inspiration. In the future, we will incorporate more omics data into our model framework to investigate the heterogeneity of cells from multi-dimensions.

### scEMAIL can be extended to annotate scATAC data

Most existing methods were designed only for annotating scRNA-seq data, thus limiting their use for other data types. In addition to the aforementioned multi-omics experiments, here we take scATAC-seq data as another example to show that scEMAIL is a flexible annotation tool and can annotate datasets of many other modalities.

scATAC-seq data reveal chromatin accessibility variations at the single-cell level, and cell-type annotation of scATAC-seq data can be used to uncover the mechanisms regulating cellular heterogeneity. However, scATAC-seq data annotation is more challenging than scRNA-seq data owing to the extreme sparsity, close-to-binary nature, and much higher dimensions [32]. To better depict the characteristics of scATAC-seq data, we replace the original ZINB distribution by the Bernoulli distribution, thereby deriving the following negative log-likelihood as the loss function:

(18) $$L_{\text{bn}}=-\frac{1}{N\cdot p}\sum_{i=1}^{N}\sum_{j=1}^{p}x_{\mathrm{ij}}\log\theta_{\mathrm{ij}}+\left(1-x_{\mathrm{ij}}\right)\log\left(1-\theta_{\mathrm{ij}}\right)$$

where $x_{\mathrm{ij}}$ is the i-th row and the j-th column of the binarized gene activity score matrix calculated from the accessibility peak matrix of scATAC-seq, and $\theta_{\mathrm{ij}}$ is the mean parameter of the Bernoulli distribution.

**Figure 10:**
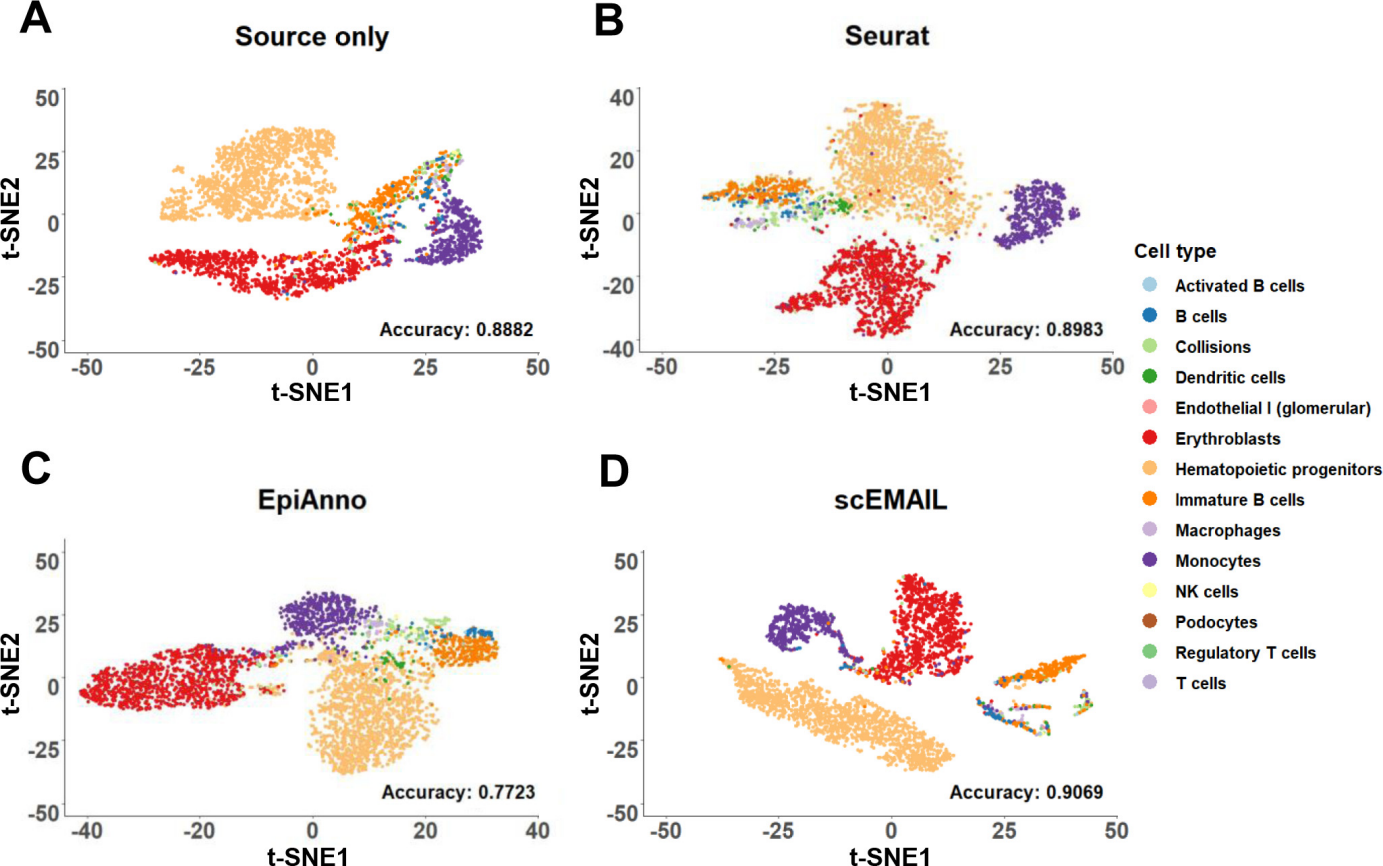
**Visualization plots of cell-type detectionbyfour compared methodsforthescATAC-seqdataset “mouse bone marrow” via t-SNE** The scATAC-seq dataset “mouse bone marrow” is derived from [33]. The 2-dimensional t-SNE projection of the latent representations and annotation accuracy from source model only (**A**), Seurat (**B**), EpiAnno (**C**), and scEMAIL (**D**) are shown. scATAC-seq, single-cell sequencing assay for transposase-accessible chromatin.

We utilized two scATAC-seq datasets of mouse bone marrow [33] for experiments. One dataset named “BoneMarrow_62216” was taken as source data, while another replicate named “BoneMarrow_62016” was taken as target data. Apart from the performance of scEMAIL and “source only”, we also showed the results of the traditional method, Seurat v3, which is also available on scATAC-seq data, and a deep learning-based method named EpiAnno [32], which is designed for the annotation of scATAC-seq data. EpiAnno is a probabilistic generative model integrated with a Bayesian neural network, and it supposes that cell embeddings follow a Gaussian mixture distribution. The t-SNE visualization plot of each method (for Seurat, the inputted embedding features were obtained by performing latent semantic indexing on the scATAC-seq peak matrix) and the corresponding annotation accuracy are shown in Figure 10. We observed that scEMAIL showed satisfactory performance in both annotation accuracy and the exploration of cell similarity. scEMAIL clearly separated the rather small cell population “immature B cells”, while the other three experiments mixed it with different cell types. EpiAnno annotated different datasets directly without model adaptation; hence, the relationship of target data may not be fully explored. The aforementioned experiments show the flexibility of the local affinity constraints module in scEMAIL to fit in other data types besides scRNA-seq data.

## Conclusion

Cell-type annotation has always been a vital part of scRNA-seq data analysis. The continuous development of sequencing technology and the ever-increasing sample size place higher demands on automatic annotation tools. An ideal method for cell annotation must be versatile, practical, and easy to operate. Accordingly, we proposed scEMAIL, a transfer learning-based algorithm for single-cell RNA annotation.

Our method is summarized in three sections, which are source model pre-training on the source data, the novel cell-type detection module to explore the existence of novel cell types and identify them, and source model fine-tuning for adaptation on the target data.

In the pre-training stage of the source model, in addition to the conventional data denoising and classification, we introduced two auxiliary classifiers to measure the consistency of model predictions. This measure was used in the novel cell-type detection module to deal with the prediction errors caused by the batch effect. Moreover, only offering the source model to users without sharing the source data is also innovative. It eliminates the access restriction of source data during model adaptation, which addresses the privacy concern and reduces the computational burden for users.

For the novel cell-type detection module, there are several contributions as follows. First of all, we did not simply use the commonly used entropy or confidence to measure the uncertainty of the predictions, but adopted the E-score, which is a comprehensive and robust uncertainty measurement scheme. Secondly, we did not need to subjectively judge the existence of new cell types in the target data, but to test the bimodality of the distribution based on their E-scores, thus to automatically detect whether a new cell type is present in the target data. In addition, our method does not need to predefine the threshold for target data partition, but applies an adaptive threshold through manifold mixup.

In the model adaptation stage, our contributions are mainly reflected in the following two aspects. First of all, unlike conventional deep learning-based annotation methods that train “known” and “unknown” cells together, our method differentiates the target cells during the training process, only training the “known” cell types, thus avoiding overconfident predictions and error propagation. Secondly, we introduce a multi-order neighborhood affinity constraint to mitigate the batch effect. Unlike the frequently used information that only considers the nearest neighbors or mutual neighbors, we also present the concept of “extensional neighbors” to collect the high-order neighborhood information of the data. It is considered to introduce more reliable self-supervised signals for different shapes of low-dimensional manifolds.

In this work, we used scRNA-seq data as a representative to illustrate our model. However, scEMAIL is not limited to scRNA-seq data, it can be generalized to other omics data through simple deformation. Some attempts on the paired CITE-seq data and scATAC-seq data in the experiment section can preliminarily illustrate this statement.

Overall speaking, our method constructs an entire framework for automatic source-free tools in cell-type annotation and novel cell-type detection. Our experimental section also establishes a standard paradigm of all the possible realistic scenarios (*i.e.*, experiments under four settings and we define an index for each setting in simulated experiments to quantify the degree of overlap between the source and target data), and builds a comprehensive benchmark for comparisons. We believe this work has a strong significance of reference for scholars who are engaged in related research.

## Code availability

The source code of scEMAIL can be accessed at https://github.com/aster-ww/scEMAIL and https://ngdc.cncb.ac.cn/biocode/tools/BT007335/releases/v1.0.

## Supplementary material

Supplementary File S1 **Supplementary notes**

Supplementary Figure S1 **Analysis of simulated experiments in “smaller” situation** Line graphs of total annotation accuracy under four settings with the variation of cluster count (**A**), “partialness” (**B**), “openness” (**C**), and “privateness” (**E**). The value of H-score is also provided for open (**D**) and open-partial (**F**) settings.

Supplementary Figure S2 **The mapping between real cell types and predicted cell types from four compared methods of dataset “mammary gland” via Sankey plots** Dataset “mammary gland” [3] is under open setting, including three target private cell types. The annotation results of scmap (**A**), scSemiCluster (**B**), scArches (**C**), and scEMAIL (**D**) are shown.

Supplementary Figure S3 **Ablation study on expert ensemble measurement of uncertainty** Histograms of H-score using ensemble as well as single (**A**) and two (**B**) measurements of uncertainty to detect novel cell types in 4 groups of real datasets under open or open-partial settings.

Supplementary Figure S4 **Ablation study on neighbor affinity constraints** Scatter plots of total annotation accuracy with and without considering bidirectional (**A**), unidirectional (**B**), and extensional (**C**) neighbors in 8 groups of real datasets.

Supplementary Figure S5 **Ablation study on target data partition** Histograms of H-score with and without data partition when performing model adaptation in 4 groups of real datasets under open or open-partial settings.

Supplementary Figure S6 **The mapping between real cell types and predicted cell types from four compared methods on the cross-tissue experiments with source tissue “limb muscle” and target tissue “diaphragm” via Sankey plots** Source tissue “limb muscle” and target tissue “diaphragm” are derived from [3]. Target tissue has two target private cell types. The annotation results of scmap (**A**), ItClust (**B**), scArches (**C**), and scEMAIL (**D**) are exhibited.

Supplementary Figure S7 **The mapping between real cell types and predicted cell types from four compared methods of the differential dataset “testis” via Sankey plots** The differential dataset “testis” is derived from [28]. For this task, the most differentiated cell type “sperm” is artificially removed in the source data. The annotation results of scmap (**A**), scSemiCluster (**B**), scArches (**C**), and scEMAIL (**D**) are exhibited. SSC, spermatogonial stem cells.

Supplementary Figure S8 **Visualization plots of cell-type detection from source model only and scEMAIL on single-omics and multi-omics experiments via t-SNE** This task is applied on the paired data from 10X 10 k PBMC dataset downloaded from 10X Genomics website (https://www.10xgenomics.com/resources/datasets/10-k-pbm-cs-from-a-healthy-donor-gene-expression-and-cell-surface-protein-3-standard-3-0-0) with cell type “CD8+ T cells” set as novel cell type. We exhibit the 2 dimensional t-SNE projection of the latent representations from source model only on single-omics (**A**) and multi-omics (**B**) data, as well as the corresponding results of scEMAIL on single-omics (**C**) and multi-omics (**D**) data.

Supplementary Table S1 **Summary of 8 scRNA-seq and 2 scATAC-seq annotation methods for comparison**

Supplementary Table S2 **Biological and statistical information of 8 groups of scRNA-seq datasets**

Supplementary Table S3 **Biological and statistical information of two atlas-level scRNA-seq datasets**

Supplementary Table S4 **Detailed selection scheme about six annotation tasks on atlas-level scRNA-seq datasets**

Supplementary Table S5 **Biological and statistical information about four annotation tasks on cross-tissue experiments**
