# Supplementary material for: scEMAIL: Universal and Source-free Annotation Method for scRNA-seq Data with Novel Cell-type Perception
Source: Genomics Proteomics Bioinformatics. 2023 Jan 3;20(5):939–58. doi: 10.1016/j.gpb.2022.12.008 (PMC10025768; doi:10.1016/j.gpb.2022.12.008)
Supplement: Supplementary File S1 — Supplementary notes [file mmc1.docx]

**File S1 Supplementary notes**

**Data preprocess**

For preprocessing, we handled the source data and target data together and aligned their gene names. The following preprocessing steps were conducted using the scanpy package, including library size normalization, data logarithmization, selection of highly variable genes, and z-score normalization. The preprocessed data was applied as autoencoder input, and we used the corresponding original count data for the zero-inflated negative binomial (ZINB) distribution modeling.

**Tests for the bimodal structure of the empirical distribution based on E-score** *Bimodality coefficient (BC)*

Based on the empirical relationship between bimodality and the third (skewness) and fourth (kurtosis) statistical moments of a distribution, the formulation of BC [1] is

$$\begin{aligned} \text{BC}=\frac{\nu_{3}^{2}+1}{\nu_{4}+3\frac{(n-1)^{2}}{\left( n-2 \right)\left( n-3 \right)}}\#(1) \end{aligned}$$

where $\nu_{3}$ indicates the skewness of the distribution and $\nu_{4}$ refers to its excess kurtosis. Both of them are corrected for sample bias [2]. This coefficient is calculated because we observe that a bimodal distribution may have an asymmetric character, very low kurtosis, or both of two. The value of BC ranges between 0 and 1. A benchmark value of approximate 0.555 is given by a uniform distribution. We compare the BC value of our empirical distribution to this benchmark. Higher number indicates bimodality while lower number suggests unimodality.

In practical use, to alleviate the potential risk for a skewed unimodal distribution been recognized erroneously as bimodal by the BC value [3], we apply square root transformation to the original E-score and then calculate the BC value of the empirical distribution after transformation.

*The Hartigan’s dip test*

The Hartigan’s dip test [4] measures the maximum difference between the empirical distribution function and the unimodal distribution function which minimizes that maximum difference. We suppose that the bimodal structure exists in target data if the test statistic is statistically significant (*P* value < 0.01).

We recorded testing results of bimodality using two different measurements for 8 pairs of real datasets in Table S6.

**Table S6 Testing results of bimodality using two different measurements for 8 pairs of real datasets**

| **Setting** | **Dataset** | ***P* value** | **The BC value** | **Result** |
| --- | --- | --- | --- | --- |
| Closed | Placenta | 0.2887 | 0.4731 | Unimodality |
|  | Bone marrow | 0.1417 | 0.3191 | Unimodality |
| Partial | Pancreas | 0.3315 | 0.4685 | Unimodality |
|  | Trachea | 0.2773 | 0.3567 | Unimodality |
| Open | Mammary gland | 0 | 0.6054 | Bimodality |
|  | Lung | 0 | 0.6092 | Bimodality |
| Open-partial | Neonatal rib | 0.0019 | 0.5942 | Bimodality |
|  | Peripheral blood | 0 | 0.6314 | Bimodality |

*Note*: The third column exhibits the *P* value of the Hartigan’s dip test (< 0.01 indicates bimodality) while the fourth column shows the calculated BC values (> 0.555 suggests bimodal structure). BC, bimodality coefficient.

**Implementation of scEMAIL**

scEMAIL was implemented in Python 3 with PyTorch. We set $p = 1000$ to select highly variable genes. The encoder network contained layer size of [1000, 256, 64, 32] to produce a latent representation of 32 dimensions and the decoder network had a symmetric structure. The network was trained by the Adam optimizer with a learning rate 0.001 to update variables and batch size $b$ equalled 256.

*Source model training*

Similar to [5], to increase the discriminability of the source model and encourage cells to lie in tight separated clusters, in practical implementation, source model was optimized with smooth label ${\overset{\sim}{q}}_{i,k}$ rather than the one-hot encoded label $q_{i,k}$, where ${\overset{\sim}{q}}_{i,k}=(1-\beta)I(y_{i}^{s}=k)+\beta/K$with the default value of $\beta=0.1$. We trained the overall $\mathcal{L}_{s}$ loss with maximum number of epochs 1000.

*Model adaptation*

The default settings of hyper-parameters $\alpha$and $M$ are 0.1 and 5, respectively. In practical implementation, we also provided a choice to incorporate pseudo label-induced self-supervised learning [6]. This self-supervised pseudo-labeling strategy can better guide the feature training of target data. Similar to the idea of weighted soft K-means clustering, we can obtain the initial centroid of each cell type in the target data via bank $\mathcal{R}$ and $\mathcal{S}$

$$\begin{aligned} \begin{aligned} \xi_{k}^{init}=\frac{\sum_{i=1}^{N_{t}} \mathcal{S}_{i,k}\cdot\mathcal{R}_{i}^{t}}{\sum_{i=1}^{N_{t}} \mathcal{S}_{i,k}},\text{ }k=1,\cdots,K\#(2) \end{aligned}\# \end{aligned}$$

here $\mathcal{S}_{i,k}$ is the $k$-th element of vector $\mathcal{S}_{i}$. Then we can assign the initial pseudo label ${\overset{^}{y}}_{i}^{t}$ of each target sample to its nearest centroid,

$$\begin{aligned} {\overset{^}{y}}_{i}^{t}=\arg\min_{k}\left( 1-\pi\left( \xi_{k}^{init},\mathcal{R}_{i} \right) \right),i=1,\cdots,N_{t}\#(3) \end{aligned}$$

The centroids and pseudo labels for the target data can hence be updated to obtain more robust and reliable results,

$$\begin{aligned} \xi_{k}=\frac{\sum_{i=1}^{N_{t}} I\left( {\overset{^}{y}}_{i}^{t}=k \right)\cdot\mathcal{R}_{i}}{\sum_{i=1}^{N_{t}} I\left( {\overset{^}{y}}_{i}^{t}=k \right)},\text{ }{\overset{^}{y}}_{i}^{t}=\arg\min_{k}\left( 1-\pi\left( \xi_{k},\mathcal{R}_{i} \right) \right)\#(4) \end{aligned}$$

These pseudo labels can offer a better supervision during model adaptation.

The pseudo label-induced self-supervised classification loss of target data is formulated as,

$$\begin{aligned} \mathcal{L}_{\text{cls}}^{t}=-\frac{1}{\left\| \mathcal{D} \right\|}\sum_{i\in\mathcal{D}} \sum_{k=1}^{K} I\left( {\overset{^}{y}}_{i}^{t}=k \right)\log p_{i,k}^{t}\#(5)\#\#\#\# \end{aligned}$$

We trained the overall loss $\mathcal{L}_{t}=\mathcal{L}_{\text{zinb}}^{t}+\mathcal{L}_{\text{nbh}}^{t}+\mathcal{L}_{\text{cls}}^{t}$ until the relative change of annotation labels was smaller than 0.001.

**Detailed information about the simulated datasets**

We utilized the community approved R package splatter [7] to generate datasets under different label space settings, cluster numbers, and mutual cluster size of source and target data. In “smaller” setting, the mean size of each cluster in the source data was 500 while the mean cluster size in the target data was 1000. In “larger” setting, the mean cluster size in the source data was 1000 while that in the target data was 500. For each dataset, we assumed that the dropout rate was about 30% (with splatter parameter: dropout.mid = 1) and the size of each cell type was arranged in a proportional sequence with a ratio of 0.8. To evaluate the performance of our method under all the possible scenarios of label space settings, we conducted the following experiments with no prior information about the category affiliation of these two datasets.

*Simulations of closed and partial settings*

In this part of simulations, no novel cell types existed in the target data. In terms of the existence of source private cell types, the experiments can be divided into closed setting and partial setting.

For experiments under closed setting, we allowed the number of cell types to range from 8 to 12 with total size of the smaller one between the two datasets ranges from 4000 to 6000.

For experiments under partial setting in Table S7, we fixed the number of common cell types between source and target data at 10, *i.e.*, target cell types equalled 10. To measure the partial degree between source and target label spaces, we defined an index “partialness” as $1-\frac{\mathcal{\|T\|}}{\|\mathcal{T}_{s}\|}$, where $\|\mathcal{T}_{s}\|$ is the total number of source cell types while $\mathcal{\|T}\|=10$ corresponds to the shared number of cell types. We increased the source private cell types from 1 to 5 so as to increase the “partialness” from 0.09 to 0.33.

**Table S7 Detailed selection scheme about source and target data for the simulated experiments under partial setting**

| **Partialness** | **Source label space** | **Source private labels** |
| --- | --- | --- |
| 0.09 | {0–11} | 5 |
| 0.17 | {0–12} | 5,6 |
| 0.23 | {0–13} | 5,6,7 |
| 0.29 | {0–14} | 5,6,7,8 |
| 0.33 | {0–15} | 5,6,7,8,9 |

*Simulations of open and open-partial settings*

For experiments under open setting, similar with those in partial settings, we fixed the number of shared cell types at 10 and defined an index “openness” as $1-\frac{\mathcal{\|T\|}}{\|\mathcal{T}_{t}\|}$, where $\mathcal{\|T\|}$ is 10 and the number of target cell types $\|\mathcal{T}_{t}\|$ is varied from 11 to 15 so as to increase the “openness” from 0.09 to 0.33. The detailed information is shown in Table S8.

**Table S8 Detailed selection scheme about source and target data for the simulated experiments under open setting**

| **Openness** | **Target label space** | **Target private labels** |
| --- | --- | --- |
| 0.09 | {0–11} | 5 |
| 0.17 | {0–12} | 5,6 |
| 0.23 | {0–13} | 5,6,7 |
| 0.29 | {0–14} | 5,6,7,8 |
| 0.33 | {0–15} | 5,6,7,8,9 |

For the most challenging open-partial setting experiments exhibited in Table S9, we defined an index “privateness” as $1-\frac{\mathcal{\|T\|}}{\|\mathcal{T}_{s}\cup\mathcal{T}_{t}\|}$ and also fixed the shared number of cell types $\mathcal{\|T\|}$ at 10. We increased the source and target private cluster numbers from 1 to 5 separately. Hence the total number of cell types $\|\mathcal{T}_{s}\cup\mathcal{T}_{t}\|$ ranges from 12 to 20 and the “privateness” index is increased from 0.17 to 0.50.

**Table S9 Detailed selection scheme about source and target data for the simulated experiments under open-partial setting**

| **Privateness** | **Common label space** | **Source private labels** | **Target private labels** |
| --- | --- | --- | --- |
| 0.17 | {0,1,2,3,4,7,8,9,10,11} | 5 | 6 |
| 0.29 | {0,1,2,3,4,9,10,11,12,13} | 5,7 | 6,8 |
| 0.38 | {0,1,2,3,4,11,12,13,14,15} | 5,7,9 | 6,8,10 |
| 0.44 | {0,1,2,3,4,13,14,15,16,17} | 5,7,9,11 | 6,8,10,12 |
| 0.50 | {0,1,2,3,4,15,16,17,18,19} | 5,7,9,11,13 | 6,8,10,12,14 |

**The threshold selection of competing methods to detect “unknown” cells for real data**

For competing methods, if novel cell types exist in the target data, we have to choose a subjective threshold to annotate certain cells as “unknown”. The implementation of scmap provides a correlation score to annotate “unknown” cells while scSemiCluster measures the entropy of each cell to detect novel cell types. For ItClust and scArches, their implementations do not provide a concrete function to distinguish novel cell types yet. For the sake of fairness, we uniformly choose entropy for uncertainty measurement for the three deep learning-based methods scSemiCluster, ItClust, and scArches.

For the threshold selection of these methods, we adopted the following strategies. We first trained each model with its default threshold (for scmap, its default threshold is 0.7; for the others, after divide $\log K$ to the entropy to transform it ranging in [0, 1], we choose the default threshold as 0.5.). If the corresponding results indicated that the threshold was too high or too low, we set 0.1 as an interval to adjust the threshold. Among five alternative thresholds, the best performing model was saved and corresponding result was recorded. We recorded the selected thresholds of each method for datasets under open and open-partial settings in Table S10.

**Table S10 The selected thresholds of each method in 4 groups of scRNA-seq datasets under open and open-partial settings**

| **Dataset** | **scmap** | **ItClust** | **scSemiCluster** | **scArches** |
| --- | --- | --- | --- | --- |
| Mammary gland | 0.7 (0.9431/0.9259) | 0.3 (0.4111/0.1207) | 0.6 (0.7494/0.5474) | 0.1 (0.4847/0.4072) |
| Lung | 0.3 (0.5743/0.5543) | 0.8 (0.3112/0.3939) | 0.5 (0.7349/0.5846) | 0.1 (0.6810/0.1049) |
| Neonatal rib | 0.5 (0.3648/0.2397) | 0.5 (0.5916/0.5902) | 0.5 (0.6803/0.6737) | 0.1 (0.4339/0.0641) |
| Peripheral blood | 0.4 (0.2119/0.1653) | 0.2 (0.2124/0.0231) | 0.8 (0.6856/0.011) | 0.2 (0.2071/0.0761) |

*Note*: We also provided the corresponding total annotation accuracy and H-score under default threshold in parentheses (total accuracy/H-score).

**Sensitivity and scalability analysis**

Our method introduces two hyper-parameters: $M$ which represents the range of nearest neighbors, and $\alpha$ which defines the affinity value of unidirectional and extensional neighbors. We first exhibited the results with $M$ ranging from 3 to 7 in Figure S9. We can find that scEMAIL was very robust to the choice of $M$. For each group of datasets, the changes of accuracy were almost five percentage points above and below the original results.


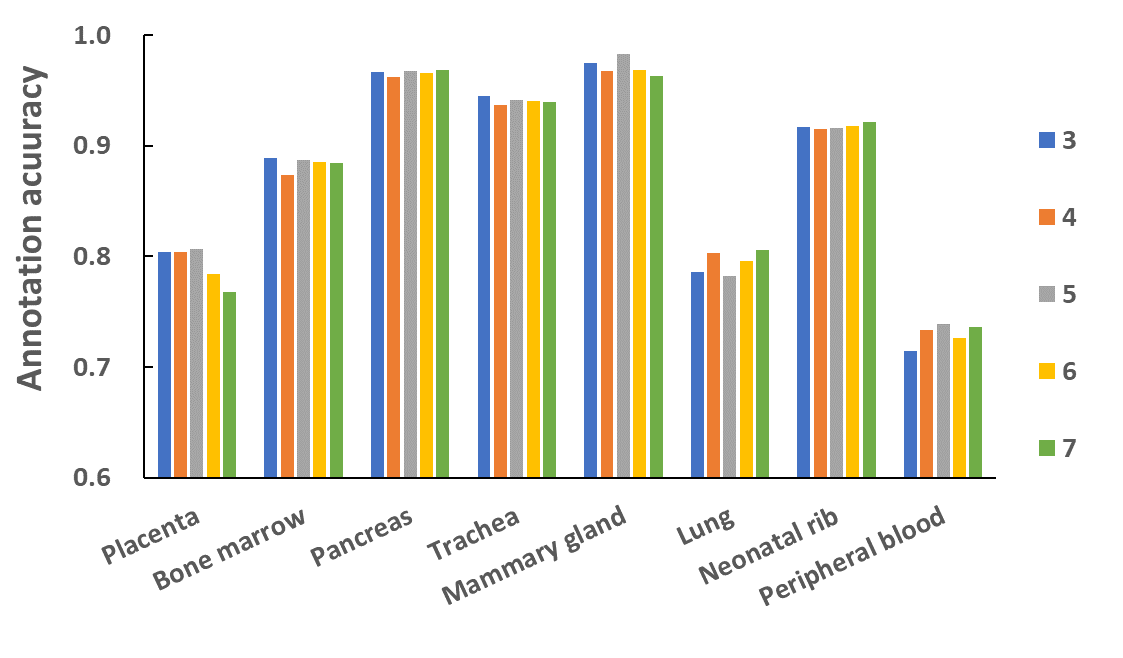


**Figure S9 Experiments on sensitivity to parameter** $\boldsymbol{M}$

Then we investigated the sensitivity of classification results when facing different $\alpha$ settings. We considered five cases of $\alpha$ in the range of ten times enlargement and ten times reductions based on our current settings ($\alpha=0.1$). Observed from Figure S10, the performance of scEMAIL was stable no matter the variation of $\alpha$. However, it was preferable to set $\alpha$ between 0.05 and 0.5, since excessive high affinity value may increase the potential risk of wrong guidance of noisy neighbors, while excessive low affinity value may not be conductive to obtain prediction consistency of these neighbors.


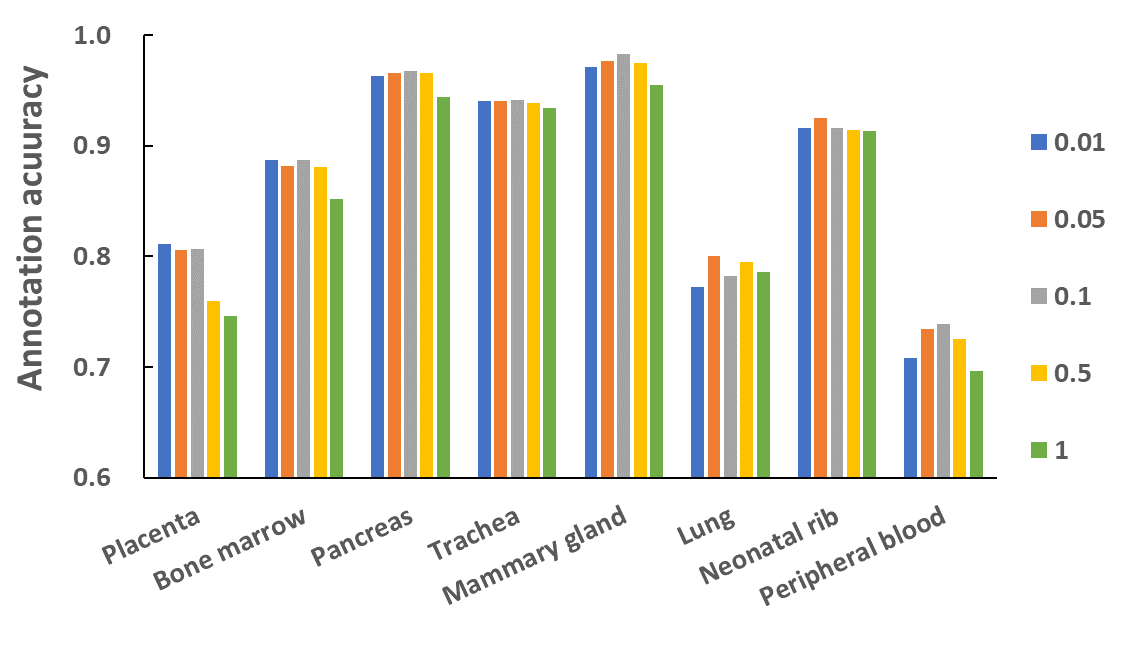


**Figure S10 Experiments on sensitivity to parameter** $\boldsymbol{\alpha}$

To meet the need of increasing scale of scRNA-seq datasets, we explored the running time and memory usage of each annotation method. We generated a series of simulated data by R package splatter with increasing sample size ranging from 5000 to 100,000. The data size indicated the individual amount of source and target data, both of them were equal in size with 2500 genes. The average elapsed time (seconds) and memory usage (GB) of all models were recorded on the same GPU server. The results in Figure S11 showed that scEMAIL can maintain a good balance between time efficiency and space efficiency, and its computational complexity approximately showed linear growth trend with the increase of sample size. Its running time was less than other methods except for ItClust and scmap and it took up much less space than ItClust, scmap, and Seurat. However, the time cost of Seurat increased most rapidly among all the conventional and deep learning-based algorithms. And ItClust required a relatively large amount of space when dealing with large-scale datasets.


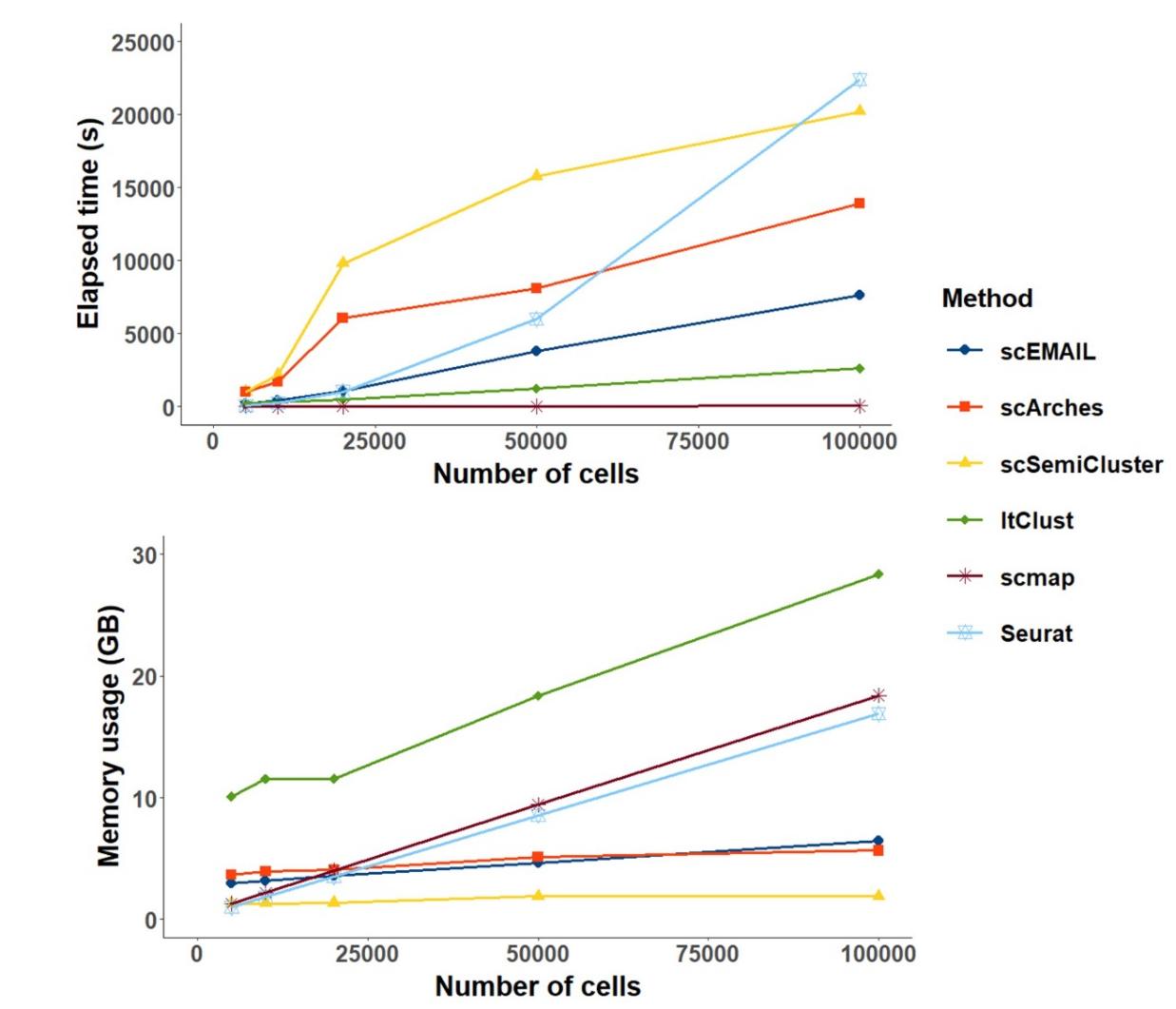


**Figure S11 Scalability experiments on large-scale simulation data**

The average elapsed time (seconds) and memory usage (GB) in various scales of source and target datasets.

We also have conducted experiments on threshold stability on dataset “neonatal rib”. Given the same source model, we assigned ten different random seeds from 1 to 10 to the target model during model adaptation, and recorded the final threshold, as well as the annotation accuracy and H-score. We can observe in Table S11 that, for ten different random seeds, the change of the final threshold does not exceed 0.03, and the change of total accuracy and H-score does not exceed 0.02. That is to say, the randomness of $\lambda_{i,j}$ will not affect the threshold largely and final annotation result is quite stable.

**Table S11 Robustness analysis of adaptive threshold via manifold mixup on dataset “neonatal rib”**

| **Seed** | **Final threshold** | **Total accuracy** | **H-score** |
| --- | --- | --- | --- |
| 1 | 0.4277 | 0.9071 | 0.9049 |
| 2 | 0.4286 | 0.9022 | 0.9010 |
| 3 | 0.4251 | 0.9129 | 0.9113 |
| 4 | 0.4266 | 0.9055 | 0.9040 |
| 5 | 0.4311 | 0.9055 | 0.9041 |
| 6 | 0.4211 | 0.9154 | 0.9136 |
| 7 | 0.4081 | 0.9162 | 0.9137 |
| 8 | 0.4286 | 0.9088 | 0.9070 |
| 9 | 0.4181 | 0.9071 | 0.9054 |
| 10 | 0.4234 | 0.9203 | 0.9179 |

**Robustness analysis of the adaptive threshold applying on target data of different heterogeneity**

The threshold we apply is data-driven, and it is adaptively and automatically decided according to different target data. If the target data contains very heterogeneous cell types, it is supposed that it tends to yield a relatively lower threshold. This is because in this case, the random cell pairs are more dissimilar and thus the patterns of manifold mixup are more novel. As a result, their corresponding E-scores as well as the generated threshold will be lower. However, we need to emphasise that the threshold value here refers to the relative threshold value (*i.e.*, the relative position of the threshold value in the E-score distribution of target data). It is meaningless to compare the absolute threshold value cross various experiments, because our classifiers include a batch normalization layer [8], which re-centers and re-scales samples within each batch and changes their distribution of soft labels.

To explore how different heterogeneity of target datasets will influence the final threshold and model performance, we have conducted a set of experiments. A differentiation dataset from the adult human testis [9] is applied because there is a clear relationship of relative similarity between cell types. The differentiation process of sperm cells follows the following precedence relationship of pseudotime: spermatogonial stem cells (SSC), differentiating S'gonia, early primary S'cytes, late primary S'cytes, round S'tids, elongated S'tids, and sperm. We can suppose that the similarity of cell types that are closer in pseudo time is stronger, while the cell types beyond the sperm cell differentiation lineage, such as leydig cell and macrophage, are not very similar to other cell types. This dataset comes from three different donors. And we filtered different cell types from donor 3 to obtain three target datasets A, B, and C. For all of them, we retained 400 sperm cells as novel cell types and 4 other cell types as common cell types with the source data, each type comprising 100 cells. For target dataset A, these four common cell types are early primary S'cytes, late primary S'cytes, round S'tids, and elongated S'tids; for target dataset B, they are early primary S'cytes, late primary S'cytes, elongated S'tids, and macrophage; for target dataset C, they are early primary S'cytes, round S'tids, leydig cell, and macrophage. We believe that the similarity of the cell types contained in A, B, and C is gradually weakened, so they represent the three levels of heterogeneity: low (A), middle (B), and high (C), respectively. We selected the cells from donors 1 and 2 as the source data (artificially removed cell type “sperm” in the source data as the novel cell type in the target data) to train the source model. The same source model was adapted on these three target datasets, and we recorded the median value of final threshold, relative threshold (the ratio of the threshold to the overall distribution), total accuracy, and the H-score with 10 random seeds in Table S12. We can see that the final relative threshold does tend to decrease slightly with increasing heterogeneity, as we discussed. However, this change does not affect the performance of the model. There are two main reasons for this phenomenon.

**Table S12 Threshold robustness analysis of scEMAIL applying on target data of different heterogeneity**

| **Heterogeneity level** | **Final threshold** | **Percentage of threshold** | **Total accuracy** | **H-score** |
| --- | --- | --- | --- | --- |
| Low | 0.4147 | 51.88% | 0.8925 | 0.8899 |
| Middle | 0.4192 | 51.19% | 0.9025 | 0.9008 |
| High | 0.4455 | 44.50% | 0.8969 | 0.8955 |

*Note*: Percentage of threshold refers to the relative position of final threshold compared to the E-scores of target data, *i.e.*, the percentage of the target data that the threshold exceeds.

First, although some cell types are similar, as long as cells have different cell-type labels, the encoder and classifiers will regard them as different cell types and try to distinguish between them during pre-training classification tasks. Therefore, regardless the level of cell-type heterogeneity, the random selection of cell pairs for manifold mixup (rather than mixup of the original input) will result in a relatively novel synthetic pattern. Therefore, the impact of large heterogeneity on the generated pattern is relatively small, and it will not cause large threshold fluctuations. Secondly, the self-supervised information introduced by the neighborhood affinity constraints during target model adaptation can assist a certain cell in ensuring its cell type and guide its soft label towards the correct direction. With the continuous training of the network, this self-corrected process could change the E-scores of target cells, especially those in the ambiguous zone. In the later stage of training, the distribution differences between the common and novel cell types will become more distinct. Their overlap will be less and less, and it will be much easier to divide them. To illustrate this point more intuitively, we visualized the results of threshold division before and after model adaptation for target data with different heterogeneity levels in Figure S12. It can be seen that after adaptation, the distribution of common and novel cell types has a large interval. The model is not sensitive to the selection of the final threshold; therefore, the small fluctuations of the final threshold will not affect the accuracy of our model.


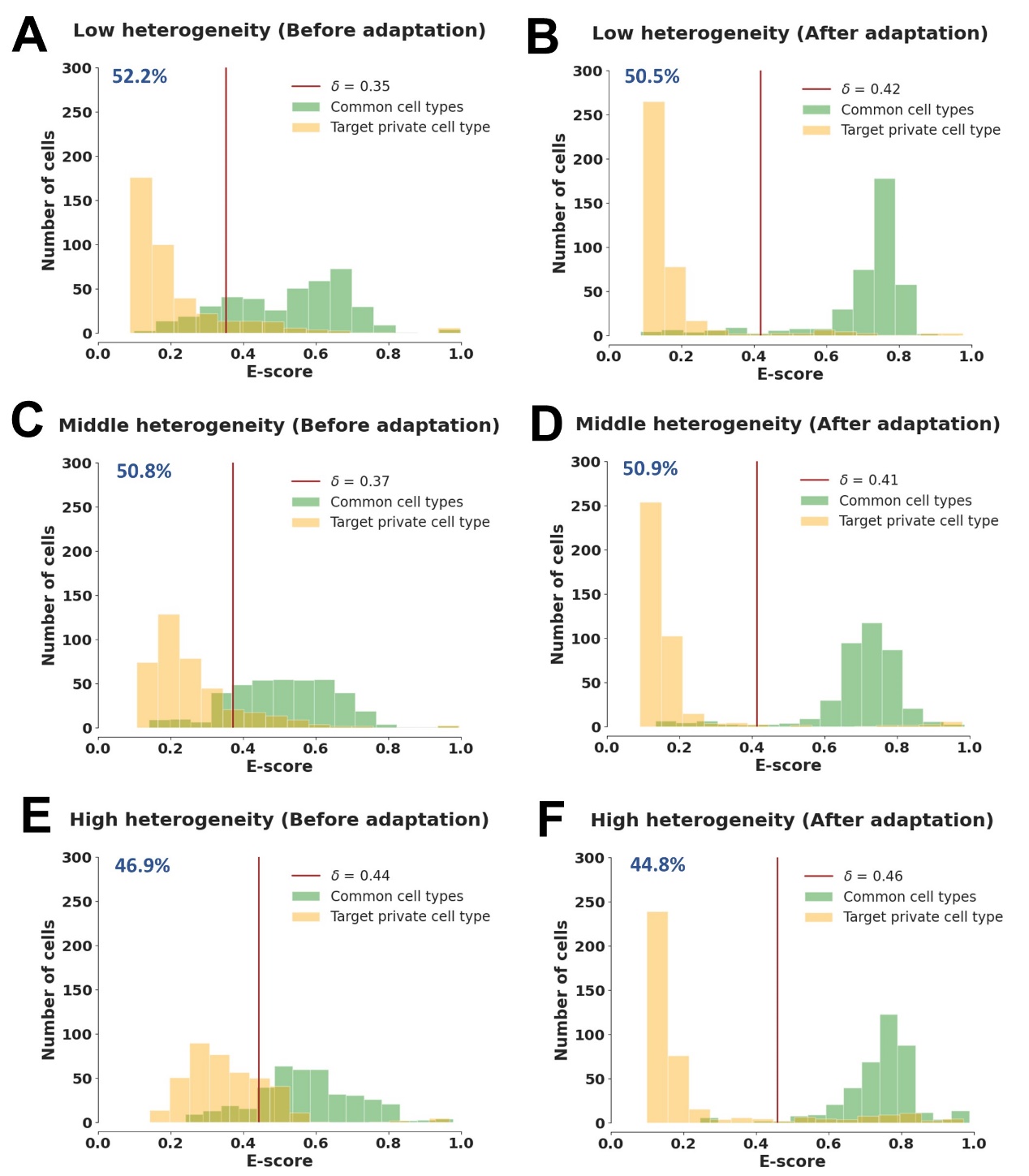


**Figure S12 The distribution difference based on E-scores before and after model adaptation of datasets “testis” via frequency histograms**

For experiments of low heterogeneity level, results of target data partition before (**A**) and after (**B**) model adaptation are exhibited, respectively. For experiments of middle heterogeneity level, results of target data partition before (**C**) and after (**D**) model adaptation level are also shown. For experiments of high heterogeneity level, we also provide results of target data partition before (**E**) and after (**F**) model adaptation. The bold vertical line is the adaptive threshold given by manifold mixup. The relative position of the threshold (the percentage of the target data that the threshold exceeds) is marked in the upper left corner of each figure.

**Discussion on the combination strategy of three uncertainty measurement**

Since there is no explicit prior information or research showing the relative importance of these three scores, our combination strategy here is to first convert them into positive indicators with a range between [0, 1]. *i.e.*, entropy needs to be converted to $1-\text{EN}/\log K$. Then we give the same weight of 1/3 to these three scores and add them together. Such a strategy is the most straightforward and easy to understand. In order to further illustrate whether it is necessary to apply other combination strategies, we conducted some exploratory experiments in four groups of scRNA-seq datasets under open and open-partial settings.

First, we have recorded the standard deviations of these three scores in the Table S13 to see whether the difference of their distribution is significant. It can be seen that the magnitudes of the three scores are not much different on the whole, and the standard deviation of confidence may be relatively smaller. Therefore, the first new combination scheme we tried was to assign weights to the three scores which is proportional to their corresponding standard deviation, which is

$$\begin{aligned} \mathrm{Weight}_{EN}=\frac{\mathrm{std}_{EN}}{\mathrm{std}_{TOTAL}}, \mathrm{Weight}_{CF}=\frac{\mathrm{std}_{CF}}{\mathrm{std}_{TOTAL}}, {\begin{aligned} \\ \mathrm{Weight} \end{aligned}}_{CS}=\frac{\mathrm{std}_{CS}}{\mathrm{std}_{TOTAL}}\#(6) \end{aligned}$$

where $\mathrm{std}_{TOTAL}=\mathrm{std}_{EN}+\mathrm{std}_{CF}+\mathrm{std}_{CS}.$

We have also tried another scheme which is from the opposite side, *i.e.*, assigning weights to the three scores which is proportional to the inverse of their corresponding standard deviation,

$$\begin{aligned} {We\mathrm{ight}}_{EN}^{'}=\frac{1/{\mathrm{std}_{EN}}}{\mathrm{std}_{TOTAL}^{'}}, {We\mathrm{ight}}_{CF}^{'}=\frac{1/{\mathrm{std}_{CF}}}{\mathrm{std}_{TOTAL}^{'}}, {We\mathrm{ight}}_{CS}^{'}=\frac{1/{\mathrm{std}_{CS}}}{\mathrm{std}_{TOTAL}^{'}}\#(7) \end{aligned}$$

where ${\mathrm{std}_{TOTAL}^{'}=1}/{\mathrm{std}_{EN}}+1/{\mathrm{std}_{CF}}+1/{\mathrm{std}_{CS}}$.

The changes of accuracy are shown in Table S13. It can be seen that, except for the dataset “lung”, the performance of scEMAIL applying these two schemes on other datasets has deteriorated a little bit. Overall, since the magnitude of the standard deviation of these three scores is relatively close, considering the factor of the standard deviation has little effect on the effect of the model.

The third attempt we made was to use the entropy weight-TOPSIS method to assign different weights to the three scores based on their distribution’s entropies. It is assumed that the distribution of a score with smaller entropy has more information and should be more important, and the corresponding result as shown in Table S13. It can be seen that the performance of model varies from dataset to dataset, but overall is not much different from directly assigning equal weights.

**Table S13 The standard deviation of three scores and results of another three combination strategies in 4 groups of scRNA-seq datasets under open and open-partial settings**

| **Dataset** | **Entropy** | **Confidence** | **Consistency** | **Proportional to std** | **Proportional to the inverse of std** | **Entropy weight-TOPSIS** |
| --- | --- | --- | --- | --- | --- | --- |
| Mammary gland | 0.3886 | 0.3000 | 0.2121 | −0.0052/  −0.0032 | −0.0020/  −0.0004 | −0.0057/  −0.0038 |
| Lung | 0.3283 | 0.3038 | 0.1965 | +0.0007/  −0.0003 | −0.0195/  −0.0157 | −0.1015/  −0.0757 |
| Neonatal rib | 0.3917 | 0.3585 | 0.2272 | −0.0189/  −0.0182 | −0.0301/  −0.0302 | +0.0037/  +0.0026 |
| Peripheral blood | 0.3655 | 0.3206 | 0.1389 | −0.0052/  −0.0032 | −0.0021/  −0.0050 | −0.0013/  +0.0005 |

*Note*: We recorded the standard deviation of three scores in the first three columns. We attempted another three combination strategies of three scores. The first was assign weights to the three scores proportional to their corresponding standard deviation. Weights were calculated proportional to the inverse of the standard deviation of three scores by the second scheme. The third combination strategy was giving three scores weights by entropy weight-TOPSIS method. The changes of total annotation accuracy and H-score are shown in the last three columns (annotation accuracy/H-score).

We believe that at present, taking equal weights of three scores after pre-processing as what we apply is a relatively reasonable and direct solution. Experiments have shown that other combination strategies cannot bring about significant performance improvements. If more reasonable results are found in follow-up research, we are willing to make further attempts and improvements on this issue.

**Performance evaluation based on tissue types**

In the experiment part, we have conducted a large number of experiments on datasets from different tissue types. We now aggregate the results of these experiments and obtain the Table S14 to show whether the performance of compared models will vary greatly on different tissues. The table exhibits the top three methods for experiments on each specific tissue type. In general, the three deep learning-based methods, scSemiCluster, scArches, and scEMAIL, show good versatility in different tissues, which may be attributed to the powerful feature extraction ability of deep neural network. The performance of ItClust on tissue types “bone marrow”, “pancreas”, as well as cross-tissue experiments is satisfactory, reflecting its potential to deal with source and target data with large differences. The similarity-based algorithm Seurat performs well on tissue types “placenta”, “bone marrow”, and “trachea”, but due to its lack of novel cell-type discovery mechanism, we have not been able to test it on more tissue types for now. scmap shows ideal annotation results on differentiation data with tissue types “mammary gland” and “testis”, indicating that it can handle continuous information of cell variation well. SingleR performs well on the atlas-level experiments of “pancreas” and “immune”. In the future, we will test SingleR, CHETAH, SingleNet, and other upcoming state-of-the-art algorithms on more various tissue types and tasks for all-sided benchmarking assessments.

**Table S14 Comprehensive assessments of compared annotation tools in a broad range of tissues, sample conditions, and applications**

| **Dataset** | **Seurat** | **SingleCellNet** | **SingleR** | **CHETAH** | **scmap** | **ItClust** | **scSemiCluster** | **scArches** | **scEMAIL** |
| --- | --- | --- | --- | --- | --- | --- | --- | --- | --- |
| Placenta | ★ | / | / | / |  |  | ★ |  | ★ |
| Bone marrow | ★ | / | / | / |  | ★ |  |  | ★ |
| Pancreas |  |  | ★ |  |  | ★ |  |  | ★ |
| Trachea | ★ | / | / | / |  |  | ★ |  | ★ |
| Mammary gland | / | / | / | / | ★ |  |  | ★ | ★ |
| Lung | / | / | / | / |  |  | ★ | ★ | ★ |
| Neonatal rib | / | / | / | / |  |  | ★ | ★ | ★ |
| Peripheral blood | / | / | / | / |  |  | ★ | ★ | ★ |
| Testis | / | / | / | / | ★ |  | ★ |  | ★ |
| Immune | / |  | ★ |  |  |  |  | ★ | ★ |
| Cross-tissues | / | / | / | / |  | ★ |  | ★ | ★ |

*Note*: For certain tissue type, we mark the top three methods with “★”. And “/” indicates that we do not test this method on this tissue type yet.

**Discussion on the annotation of novel cell types**

This work focuses on how to accurately annotate those “known” cell types and recognize “unknown” cell types. Annotating specific cell-types of “unknown” cells is beside the point of this work, but it is one of the directions of our future work. Generally speaking, when the amounts of novel cells are not very large, we can choose the traditional method based on marker genes to annotate novel cell types. As mentioned in the introduction, we can first cluster these cells without supervision, and then find the corresponding marker genes for each cluster, and finally annotate the cells according to their Gene Ontology functions. However, scalable and automatic annotation methods await our follow-up research.

**References**

[1] Freeman JB, Dale R. Assessing bimodality to detect the presence of a dual cognitive process. Behav Res Methods 2013;45:83–97.

[2] Joanes DN, Gill CA. Comparing measures of sample skewness and kurtosis. J R Stat Soc: Ser D (The Statistician) 1998;47:183–9.

[3] Pfister R, Schwarz KA, Janczyk M, Dale R, Freeman JB. Good things peak in pairs: a note on the bimodality coefficient. Front Psychol 2013;4:700.

[4] Hartigan JA, Hartigan PM. The dip test of unimodality. Ann Stat 1985;13:70–84.

[5] Müller R, Kornblith S, Hinton GE. When does label smoothing help? Proc 33st Int Conf Neural Inf Process Syst 2019:4696–705.

[6] Liang J, Hu D, Feng J. Do we really need to access the source data? source hypothesis transfer for unsupervised domain adaptation. Proc 37st Int Conf Mach Learn 2020:6028–39.

[7] Zappia L, Phipson B, Oshlack A. Splatter: simulation of single-cell RNA sequencing data. Genome Biol 2017;18:174.

[8] Ioffe S, Szegedy C. Batch normalization: Accelerating deep network training by reducing internal covariate shift. Proc 32st Int Conf Mach Learn 2015:448–56.

[9] Guo J, Grow EJ, Mlcochova H, Maher GJ, Lindskog C, Nie X, et al. The adult human testis transcriptional cell atlas. Cell Res 2018;28:1141–57.
