# Supplementary figures and images for: scEMAIL: Universal and Source-free Annotation Method for scRNA-seq Data with Novel Cell-type Perception
Source: Genomics Proteomics Bioinformatics. 2023 Jan 3;20(5):939–58. doi: 10.1016/j.gpb.2022.12.008 (PMC10025768; doi:10.1016/j.gpb.2022.12.008)

## Slide 1
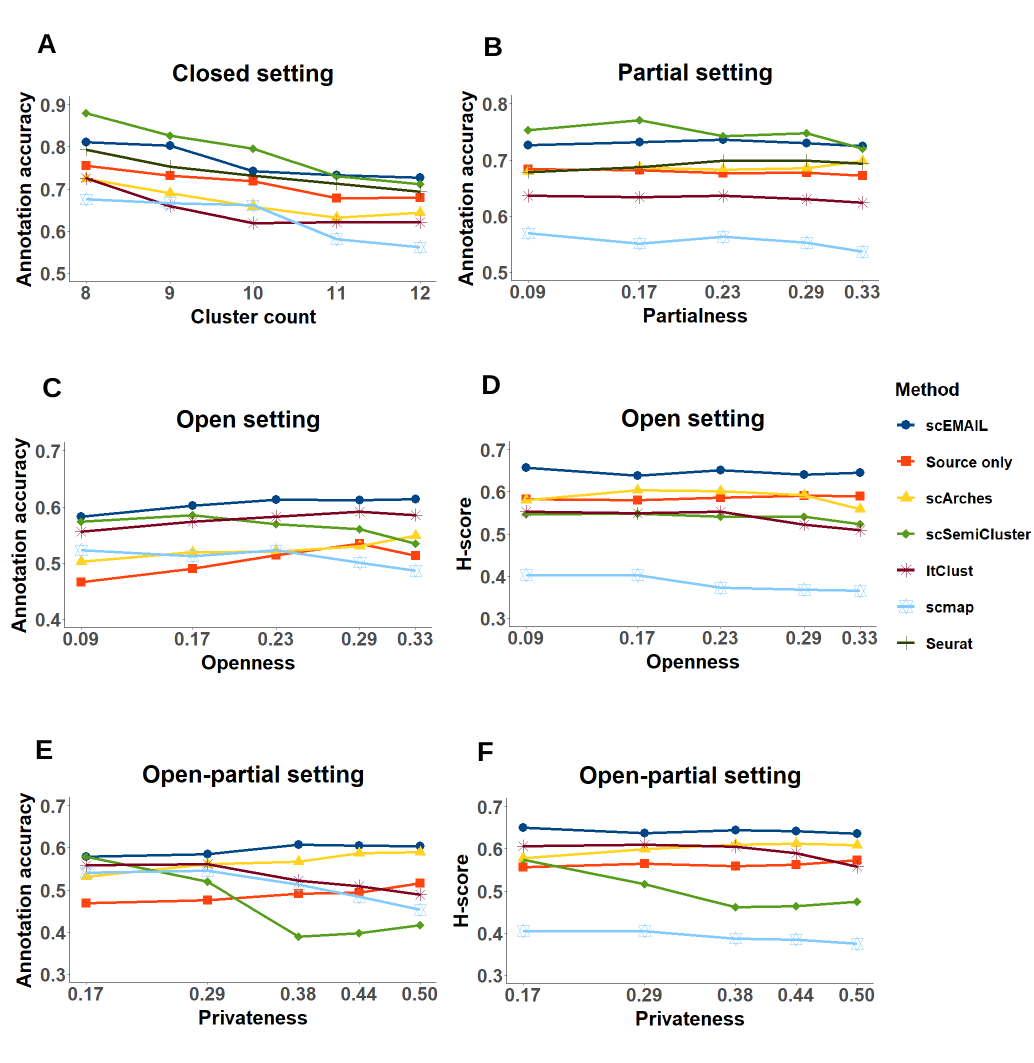

B
# A
D
C
E
F

Supplement: Supplementary Figure S1 — Analysis of simulated experiments in “smaller” situation Line graphs of total annotation accuracy under four settings with the variation of cluster count (A), “partialness” (B), “openness” (C), and “privateness” (E). The value of H-score is also provided for open (D) and open-partial (F) settings. [file mmc2.pptx]

## Slide 1
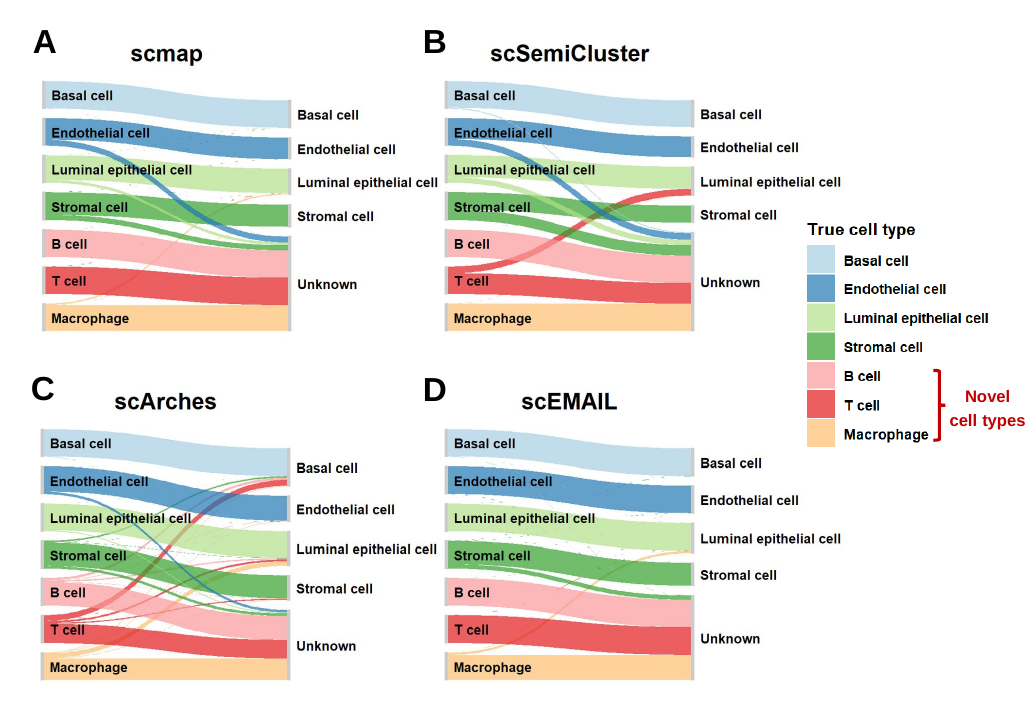

B
# A
D
C
Novel
cell types

Supplement: Supplementary Figure S2 — The mapping between real cell types and predicted cell types from four compared methods of dataset “mammary gland” via Sankey plots Dataset “mammary gland” [3] is under open setting, including three target private cell types. The annotation results of scmap (A), scSemiCluster (B), scArches (C), and scEMAIL (D) are shown. [file mmc3.pptx]

## Slide 1
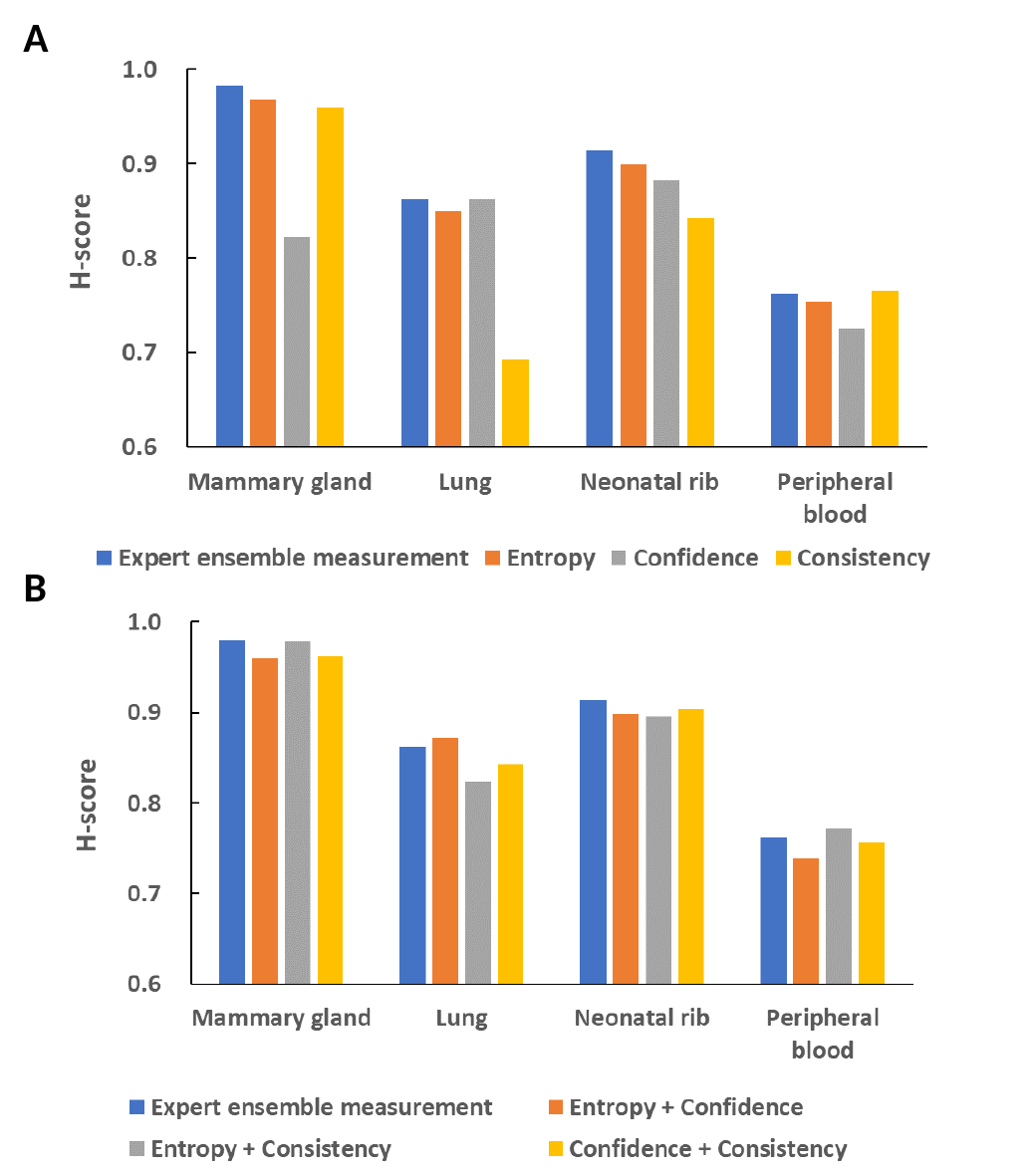

A
#
B

Supplement: Supplementary Figure S3 — Ablation study on expert ensemble measurement of uncertainty Histograms of H-score using ensemble as well as single (A) and two (B) measurements of uncertainty to detect novel cell types in 4 groups of real datasets under open or open-partial settings. [file mmc4.pptx]

## Slide 1
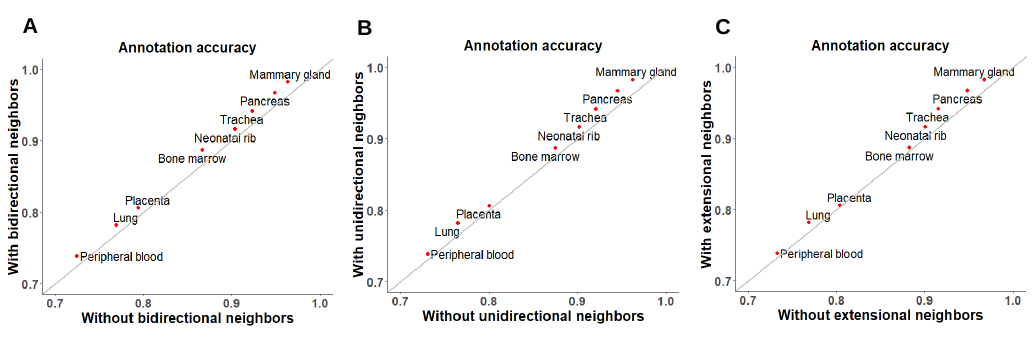

C
B
# A

Supplement: Supplementary Figure S4 — Ablation study on neighbor affinity constraints Scatter plots of total annotation accuracy with and without considering bidirectional (A), unidirectional (B), and extensional (C) neighbors in 8 groups of real datasets. [file mmc5.pptx]

## Slide 1
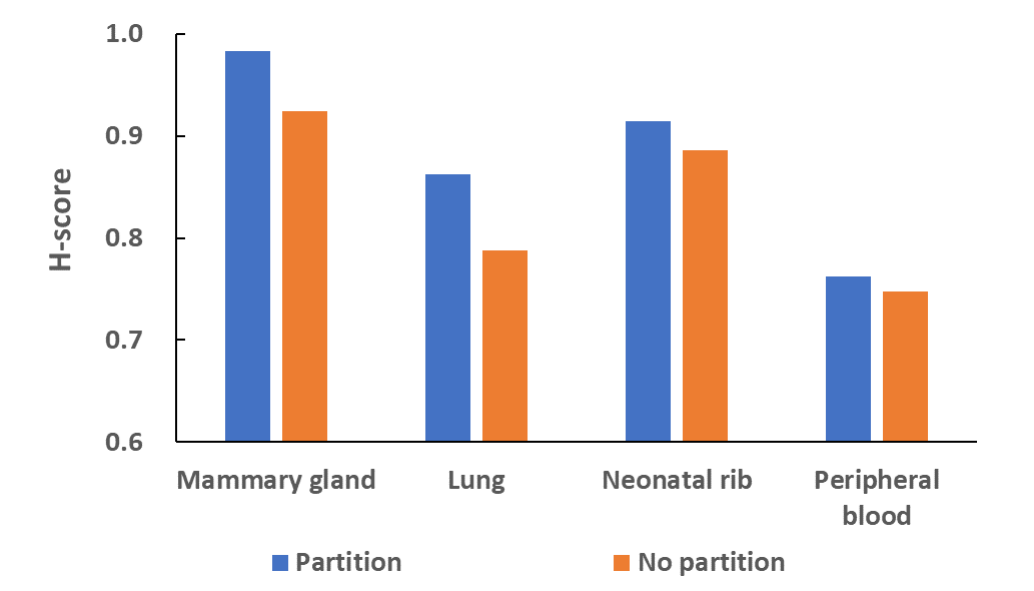

#

Supplement: Supplementary Figure S5 — Ablation study on target data partition Histograms of H-score with and without data partition when performing model adaptation in 4 groups of real datasets under open or open-partial settings. [file mmc6.pptx]

## Slide 1
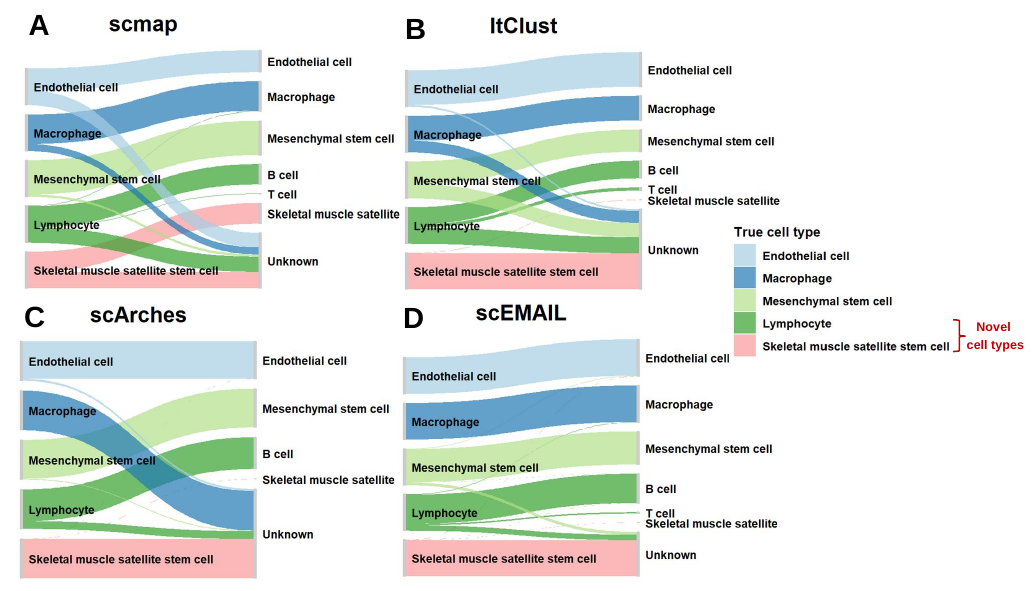

# A
B
Novel
cell types
C
D

Supplement: Supplementary Figure S6 — The mapping between real cell types and predicted cell types from four compared methods on the cross-tissue experiments with source tissue “limb muscle” and target tissue “diaphragm” via Sankey plots Source tissue “limb muscle” and target tissue “diaphragm” are derived from [3]. Target tissue has two target private cell types. The annotation results of scmap (A), ItClust (B), scArches (C), and scEMAIL (D) are exhibited. [file mmc7.pptx]

## Slide 1
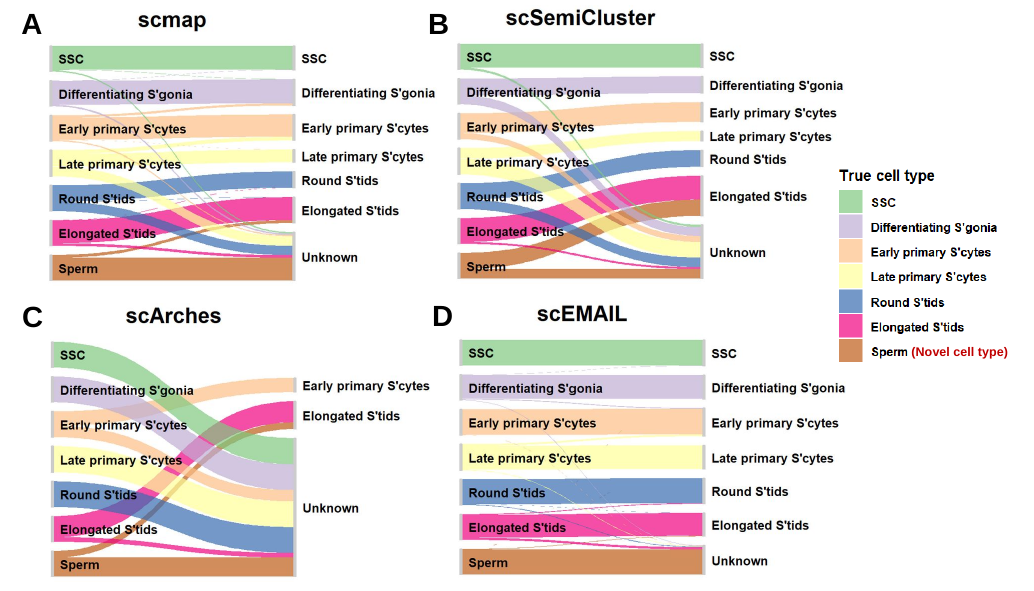

A
B
D
C
(Novel cell type)

Supplement: Supplementary Figure S7 — The mapping between real cell types and predicted cell types from four compared methods of the differential dataset “testis” via Sankey plots The differential dataset “testis” is derived from [28]. For this task, the most differentiated cell type “sperm” is artificially removed in the source data. The annotation results of scmap (A), scSemiCluster (B), scArches (C), and scEMAIL (D) are exhibited. SSC, spermatogonial stem cells. [file mmc8.pptx]

## Slide 1
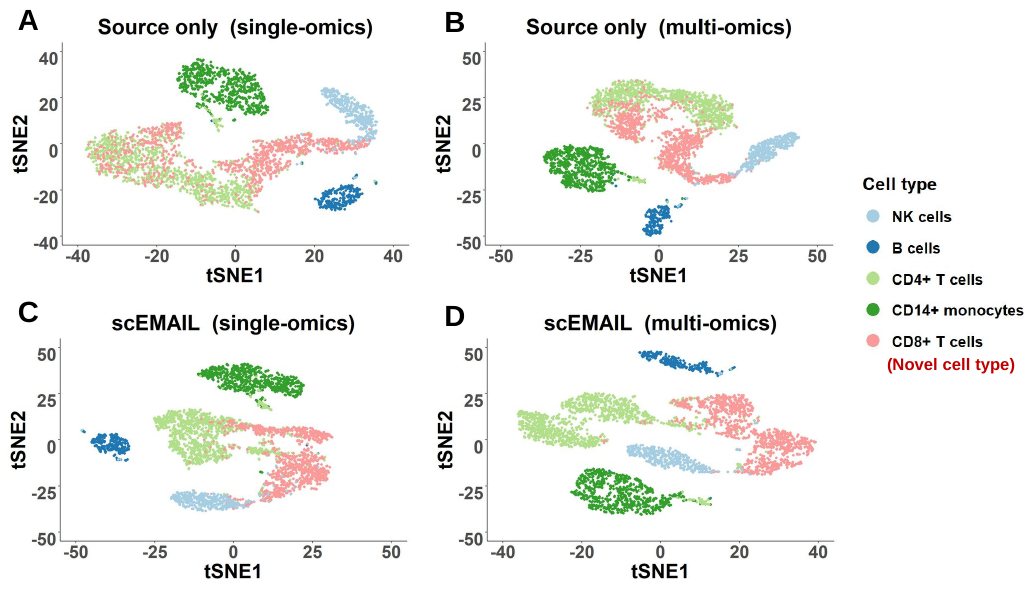

A
B
C
D
(Novel cell type)

Supplement: Supplementary Figure S8 — Visualization plots of cell-type detection from source model only and scEMAIL on single-omics and multi-omics experiments via t-SNE This task is applied on the paired data from 10X 10 k PBMC dataset downloaded from 10X Genomics website (https://www.10xgenomics.com/resources/datasets/10-k-pbm-cs-from-a-healthy-donor-gene-expression-and-cell-surface-protein-3-standard-3-0-0) with cell type “CD8+ T cells” set as novel cell type. We exhibit the 2 dimensional t-SNE projection of the latent representations from source model only on single-omics (A) and multi-omics (B) data, as well as the corresponding results of scEMAIL on single-omics (C) and multi-omics (D) data. [file mmc9.pptx]
