# Supplementary material for: scEMAIL: Universal and Source-free Annotation Method for scRNA-seq Data with Novel Cell-type Perception
Source: Genomics Proteomics Bioinformatics. 2023 Jan 3;20(5):939–58. doi: 10.1016/j.gpb.2022.12.008 (PMC10025768; doi:10.1016/j.gpb.2022.12.008)
Supplement: Supplementary Table S1 — Summary of 8 scRNA-seq and 2 scATAC-seq annotation methods for comparison [file mmc10.docx]

**Table S1 Summary of 8 scRNA-seq and 2 scATAC-seq annotation methods for comparision**

| **Method** | **Data type** | **Year** | | **Programminglanguage** | **Download uniform resource locator** |
| --- | --- | --- | --- | --- | --- |
| SingleR | scRNA-seq | 2019 | | R | https://bioconductor.org/packages/release/bioc/  html/SingleR.html |
| CHETAH | scRNA-seq | 2019 | | R | https://www.bioconductor.org/packages/release/bioc/html/CHETAH.html |
| SingleCellNet | scRNA-seq | 2019 | | R/Python | https://github.com/pcahan1/singleCellNet |
| Seurat v3 | scRNA-seq | 2019 | | R | https://github.com/satijalab/seurat |
| scmap | scRNA-seq | 2018 | | R | https://github.com/hemberg-lab/scmap |
| ItClust | scRNA-seq | 2020 | | Python | https://github.com/jianhuupenn/ItClust |
| scSemiCluster | scRNA-seq | | 2021 | Python | https://github.com/xuebaliang/scSemiCluster |
| scArches | scRNA-seq | 2022 | | Python | https://github.com/theislab/scarches |
| Seurat v3 | scATAC-seq | 2019 | | R | https://satijalab.org/signac/articles |
| EpiAnno | scATAC-seq | 2022 | | Python | https://github.com/xy-chen16/EpiAnno |
