# Supplementary material for: scEMAIL: Universal and Source-free Annotation Method for scRNA-seq Data with Novel Cell-type Perception
Source: Genomics Proteomics Bioinformatics. 2023 Jan 3;20(5):939–58. doi: 10.1016/j.gpb.2022.12.008 (PMC10025768; doi:10.1016/j.gpb.2022.12.008)
Supplement: Supplementary Table S2 — Biological and statistical information of 8 groups of scRNA-seq datasets [file mmc11.docx]

**Table S2 Biological and statistical information of 8 groups of scRNA-seq datasets**

| **Group** | **Tissue** | **Species** | **Setting** | **Platform** | **Cell types (Common/Private)** | **Cells** | | **PubMed ID** |
| --- | --- | --- | --- | --- | --- | --- | --- | --- |
| 1 | Placenta | Mouse | Closed | Microwell-seq | 23 (23/0) | 2473 | | 29474909 |
|  |  |  |  | Microwell-seq | 23 (23/0) | 1873 | | 29474909 |
| 2 | Bone marrow | Mouse | Closed | Microwell-seq | 9 (9/0) | 8166 | | 29474909 |
|  |  |  |  | Microwell-seq | 9 (9/0) | 13,019 | | 29474909 |
| 3 | Pancreas | Human | Partial | CEL-seq2 | 9 (6/3) | 2122 | | 27693023 |
|  |  |  |  | Smart-seq2 | 6 (6/0) | 2282 | | 28965763 |
| 4 | Trachea | Mouse | Partial | 10X | 5 (4/1) | 11,269 | | 30283141 |
|  |  |  |  | Smart-seq2 | 4 (4/0) | 1350 | | 30283141 |
| 5 | Mammary gland | Mouse | Open | Smart-seq2 | 4 (4/0) | 2405 | | 30283141 |
|  |  |  |  | 10X | 7 (4/3) | 4481 | | 30283141 |
| 6 | Lung | Mouse | Open | Microwell-seq | 22 (22/0) | 2512 | | 29474909 |
|  |  |  |  | Microwell-seq | 23 (22/1) | 1414 | | 29474909 |
| 7 | Neonatal rib | Mouse | Open-partial | Microwell-seq | 13 (12/1) | 1963 | | 29474909 |
|  |  |  |  | Microwell-seq | 13 (12/1) | 1217 | | 29474909 |
| 8 | Peripheral blood | Human | Open-partial | Microwell-seq | 7 (6/1) | 2719 | 32214235 | |
|  |  |  |  | Microwell-seq | 10 (6/4) | 5296 | 32214235 | |

*Note*: We name each group after their corresponding tissues. For each group, the first line exhibits the information of source data, while the second line is about the target data. For group 1, 2, 6, 7, and 8with source and target data acquired from the same platform and tissue, their batch effect come from different donors or experiment batches. In addition, to accomplish various experiment settings that meet our requirement, we artificially remove “alveolar macrophage” in the original source dataset of group 6 and “cartilage cell” in the original source dataset of group 7to obtain the source datasets that we actual take into use.
