# Supplementary material for: scEMAIL: Universal and Source-free Annotation Method for scRNA-seq Data with Novel Cell-type Perception
Source: Genomics Proteomics Bioinformatics. 2023 Jan 3;20(5):939–58. doi: 10.1016/j.gpb.2022.12.008 (PMC10025768; doi:10.1016/j.gpb.2022.12.008)
Supplement: Supplementary Table S3 — Biological and statistical information of two atlas-level scRNA-seq datasets [file mmc12.docx]

**Table S3 Biological and statistical information of two atlas-level scRNA-seq datasets**

| **Dataset** | **Cells** | **Cell types** | **Batches** | **Size of each batch** | **PubMed ID** |
| --- | --- | --- | --- | --- | --- |
| Human pancreas | 16,382 | 14 | 9 batches | celseq (1004), celseq2 (2285), fluidigmc1 (638), inDrop1 (1937), inDrop2 (1724), inDrop3 (3605), inDrop4 (1303), smarter (1492), smartseq2 (2394) | 34949812 |
| Human immune | 33,506 | 16 | 10 donors | 10X (10,727), Freytag (3347), Oetjen_A (2586), Oetjen_P (3265), Oetjen_U (3730), Villani (1022), Sun_sample1_CS (1725), Sun_sample2_KC (2281), Sun_sample3_TB (2403), Sun_sample4_TC (2420) | 34949812 |
