# Supplementary material for: scEMAIL: Universal and Source-free Annotation Method for scRNA-seq Data with Novel Cell-type Perception
Source: Genomics Proteomics Bioinformatics. 2023 Jan 3;20(5):939–58. doi: 10.1016/j.gpb.2022.12.008 (PMC10025768; doi:10.1016/j.gpb.2022.12.008)
Supplement: Supplementary Table S4 — Detailed selection scheme about six annotation tasks on atlas-level scRNA-seq datasets [file mmc13.docx]

**Table S4 Detailed selection scheme about six annotation tasks on atlas-level scRNA-seq datasets**

| **Task** | **Batches of source data** | **Batches of target data** | **Source private cell types** | **Target private cell types** | **Common cell types** |
| --- | --- | --- | --- | --- | --- |
| Human pancreas (subset) | inDrop1, inDrop2, inDrop3 | celseq, celseq2, smartseq2, fluidigmc1 | 1 | 0 | 13 |
| Human pancreas (full) | inDrop1, inDrop2, inDrop3, inDrop4 | celseq, celseq2, fluidigmc1, smarter, smartseq2 | 1 | 0 | 13 |
| Human pancreas (Source data exclude “alpha”) | inDrop1, inDrop2, inDrop3, inDrop4 | celseq, celseq2, fluidigmc1, smarter, smartseq2 | 1 | 1 | 12 |
| Human pancreas (Source data exclude “acinar”) | inDrop1, inDrop2, inDrop3, inDrop4 | celseq, celseq2, fluidigmc1, smarter, smartseq2 | 1 | 1 | 12 |
| Human immune (subset) | 10X, Freytag | Oetjen_A, Oetjen_P, Oetjen_U, Villani | 0 | 4 | 12 |
| Human immune (full) | 10X, Freytag, Sun_sample1_CS, Sun_sample2_KC | Oetjen_A, Oetjen_P, Oetjen_U, Villani, Sun_sample3_TB, Sun_sample4_TC | 0 | 4 | 12 |
