# Supplementary material for: scEMAIL: Universal and Source-free Annotation Method for scRNA-seq Data with Novel Cell-type Perception
Source: Genomics Proteomics Bioinformatics. 2023 Jan 3;20(5):939–58. doi: 10.1016/j.gpb.2022.12.008 (PMC10025768; doi:10.1016/j.gpb.2022.12.008)
Supplement: Supplementary Table S5 — Biological and statistical information about four annotation tasks on cross-tissue experiments [file mmc14.docx]

**Table S5 Biological and statistical information about four annotation tasks on cross-tissue experiments**

| **Source tissue** | **Target tissue** | **Source private cell types** | **Common cell types** | **Target private cell types** |
| --- | --- | --- | --- | --- |
| Mammary gland | Fat | basal cell, luminal epithelial cell of mammary gland, macrophage, stromal cell (4) | B cell, T cell, endothelial cell (3) | mesenchymal stem cell of adipose, myeloid cell, natural killer cell (3) |
|  | Limb muscle | basal cell, luminal epithelial cell of mammary gland, stromal cell (3) | B cell, T cell, endothelial cell, macrophage (4) | mesenchymal stem cell, skeletal muscle satellite cell (2) |
| Limb muscle | Kidney | B cell, T cell, mesenchymal stem cell, skeletal muscle satellite cell (4) | endothelial cell, macrophage (2) | epithelial cell of proximal tubule, kidney collecting duct epithelial cell, leukocyte (3) |
|  | Diaphragm | B cell, T cell, skeletal muscle satellite cell (3) | endothelial cell, macrophage, mesenchymal stem cell (3) | lymphocyte, skeletal muscle satellite stem cell (2) |

*Note*: The platforms of source and target tissues are 10X and Smart-seq2, respectively.
